# Targeting CBX3 with a Dual BET/PLK1 Inhibitor Enhances the Antitumor Efficacy of CDK4/6 Inhibitors in Prostate Cancer

**DOI:** 10.1002/advs.202302368

**Published:** 2023-11-10

**Authors:** Huaiyuan Liang, Chunguang Yang, Ruijiang Zeng, Yingqiu Song, Jianxi Wang, Wei Xiong, Binyuan Yan, Xin Jin

**Affiliations:** ^1^ Department of Urology The Second Xiangya Hospital Central South University Changsha Hunan 410011 China; ^2^ Uro‐Oncology Institute of Central South University Changsha Hunan 410011 China; ^3^ Department of Urology Tongji Hospital Tongji Medical College Huazhong University of Science and Technology Wuhan 430030 China; ^4^ Cancer center Union Hospital Tongji Medical College Huazhong University of Science and Technology Wuhan 430022 China; ^5^ Department of Urology The Third Hospital of Changsha Changsha Hunan 410011 China; ^6^ Department of Urology Pelvic Floor Disorders Center The Seventh Affiliated Hospital Sun Yat‐sen University Shenzhen 518107 China

**Keywords:** BET inhibitors, CBX3, CDK4/6 inhibitors, CRPC, PLK1

## Abstract

The development of castration‐resistant prostate cancer (CRPC) is a significant factor that reduces life expectancy among patients with prostate cancer. Previously, it is reported that CDK4/6 inhibitors can overcome the resistance of CRPC to BET inhibitors by destabilizing BRD4, suggesting that the combination of CDK4/6 inhibitors and BET inhibitors is a promising approach for treating CRPC. In this study, candidates that affect the combined antitumor effect of CDK4/6 inhibitors and BET inhibitors on CRPC is aimed to examine. The data demonstrates that CBX3 is abnormally upregulated in CDK4/6 inhibitors‐resistant cells. CBX3 is almost positively correlated with the cell cycle in multiple malignancies and is downregulated by BET inhibitors. Mechanistically, it is showed that CBX3 is transcriptionally upregulated by BRD4 in CRPC cells. Moreover, it is demonstrated that CBX3 modulated the sensitivity of CRPC to CDK4/6 inhibitors by binding with RB1 to release E2F1. Furthermore, it is revealed that PLK1 phosphorylated CBX3 to enhance the interaction between RB1 and CBX3, and desensitize CRPC cells to CDK4/6 inhibitors. Given that BRD4 regulates CBX3 expression and PLK1 affects the binding between RB1 and CBX3, it is proposed that a dual BRD4/PLK1 inhibitor can increase the sensitivity of CRPC cells to CDK4/6 inhibitors partially through CBX3.

## Graphical abstract

The findings show that CBX3 is transcriptionally upregulated by BRD4 in castration‐resistant prostate cancer (CRPC) cells. Then, it is reveal that PLK1 phosphorylated CBX3 binds with RB1 to release E2F1, which decreases the sensitivity of CRPC to CDK4/6 inhibitors. Thus, the findings propose that a dual BRD4/PLK1 inhibitor can increase the sensitivity to CDK4/6 inhibitors partially through CBX3 in CRPC cells.

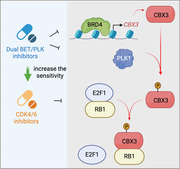

## 1 Introduction

Prostate cancer (PC) is the second most common malignancy in men and accounts for 1–2% of deaths in worldwide.^[^ ^1^, ^2^ ^]^ At present, most individuals suffering from PC are treated locally with radical prostatectomy, interstitial brachytherapy, or external beam radiotherapy alone or in conjunction with adjuvant androgen deprivation therapy (ADT).^[^ ^2^, ^3^ ^]^ It is well known that the growth of PC depends on androgen.^[^ ^4^ ^]^ ADT is the backbone of systemic therapy for advanced PC patients.^[^ ^4^ ^]^ After starting ADT, it typically takes ≈2 to 3 years for patients to develop castration‐resistant prostate cancer (CRPC).^[^ ^2^, ^3^ ^]^ Serial increases in PSA levels even after achieving castration levels of testosterone and/or evidence of disease progression shown by imaging studies are used to define CRPC.^[^ ^5^ ^]^ In recent years, potent androgen receptor (AR) pathway inhibitors (ARPIs) have been used to treat CRPC patients.^[^ ^6^ ^]^ Although CRPC patients have a clinically significant responses to ARPIs, subsequent castration resistance occurs, mainly through ligand‐dependent mechanisms and ligand‐independent mechanisms in AR signaling reactivation.^[^ ^7^ ^]^ Thus, new drugs are still needed for the treatment of CRPC.

The bromodomain and extraterminal (BET) family of chromatin readers, which include bromodomain‐containing protein (BRD) 2, BRD3, and BRD4, is currently being examined as a promising therapeutic target in the clinical treatment of CRPC patients.^[^ ^8^ ^]^ BET proteins are epigenetic readers that regulate gene expression and are involved in cancer pathogenesis.^[^ ^9^ ^]^ There is growing evidence to suggest that BRD4 plays a role in various biological processes, such as cell cycle progression, cellular proliferation, the DNA damage response, and autophagy.^[^ ^10^, ^11^ ^]^ Notably, in CRPC, BRD4 is a crucial coregulator of AR.^[^ ^12^ ^]^ Research has shown that CRPC cells, which show high AR signal transduction activity, are sensitive to BET inhibition.^[^ ^13^ ^]^ BET inhibitors (BETi) have been shown to block AR signaling and inhibit the growth of CRPC in patient‐derived models by regulating alternative splicing, resulting in reduced AR splicing and AR‐V7 expression.^[^ ^12^ ^]^ This finding suggests that BET inhibitors have therapeutic potential in the treatment of CRPC.

Furthermore, androgen has been shown to increase the translation of D‐type cyclins and induce the mRNA expression of p21Cip1 via the mammalian target of rapamycin (mTOR) signaling pathway.^[^ ^14^ ^]^ These inductive events work together to promote the formation of an active complex of D‐cyclins, p21Cip1 and cyclin‐dependent kinases 4/6 (CDK4/6), which are critical for cell cycle progression.^[^ ^14^ ^]^ Based on the traditional perspective of the cell cycle initiation, the G1‐to‐S‐phase transition is principally driven by D‐type cyclins, such as cyclin D1, cyclin D2, and cyclin D3.^[^ ^15^ ^]^ The activity of D‐type cyclins and CDK4/6 is closely linked to multiple pathways related to cell cycle initiation and progression.^[^ ^16^ ^]^ CDK4/6 inhibitors which includes palbociclib, pibociclib, and abemaciclib, represent significant advances in cancer treatment.^[^ ^15^ ^]^ Recently, there have been successful preclinical trials demonstrating the efficacy of using CDK4/6 inhibitors as a standalone treatment for PC, and a clinical trial has been carried out to evaluate the effectiveness of CDK4/6 inhibitors alone in men diagnosed with localized PC or in combination with docetaxel and prednisone for CRPC patients.^[^ ^17^ ^]^ Moreover, it has been reported that CDK4/6 inhibitors enhanced the effect of ADT and PARP inhibitors in a preclinical model of PC.^[^ ^18^ ^]^ Thus, CDK4/6 inhibitors alone or combined with other drugs are suitable options for CRPC treatment.

We previously reported that CDK4/6 inhibitors overcome the resistance of CRPC to BET inhibitors by destabilizing BRD4,^[^ ^10^ ^]^ which indicates that combination therapy involving CDK4/6 inhibitors and BET inhibitors holds significant promise for effectively treating CRPC. In this study, we aimed to examine candidates that affect the antitumor effect of CDK4/6 inhibitors and BET inhibitors on CRPC. We showed that chromobox protein homolog 3 (CBX3) was abnormally upregulated in CDK4/6 inhibitor‐resistant cells. And CBX3 was also found almost positively correlated with the cell cycle in multiple malignancies and was downregulated by BET inhibitors. Then, we demonstrated that CBX3 was transcriptionally regulated by BRD4 and modulated the sensitivity of CRPC to CDK4/6 inhibitors in an RB1‐dependent manner. We also found that CBX3 regulated PC cell cycle‐related genes through the RB1/E2F1 signaling pathway. Moreover, the experimental results showed that Polo‐like kinase 1 (PLK1)‐mediated phosphorylation of CBX3 promoted CBX3 binding with RB1 and desensitized CRPC cells to CDK4/6 inhibitors. Given that BRD4 regulates CBX3 expression and PLK1 affects the binding of RB1 and CBX3, our results demonstrate that dual BET/PLK1 inhibitors enhance the sensitivity of CRPC to CDK4/6 inhibitors partially through the CBX3/RB1 axis.

## 2 Results and Discussion

### 2.1 The Cell Cycle‐Associated Gene CBX3 is Directly Regulated by BRD4

**Figure 1:**
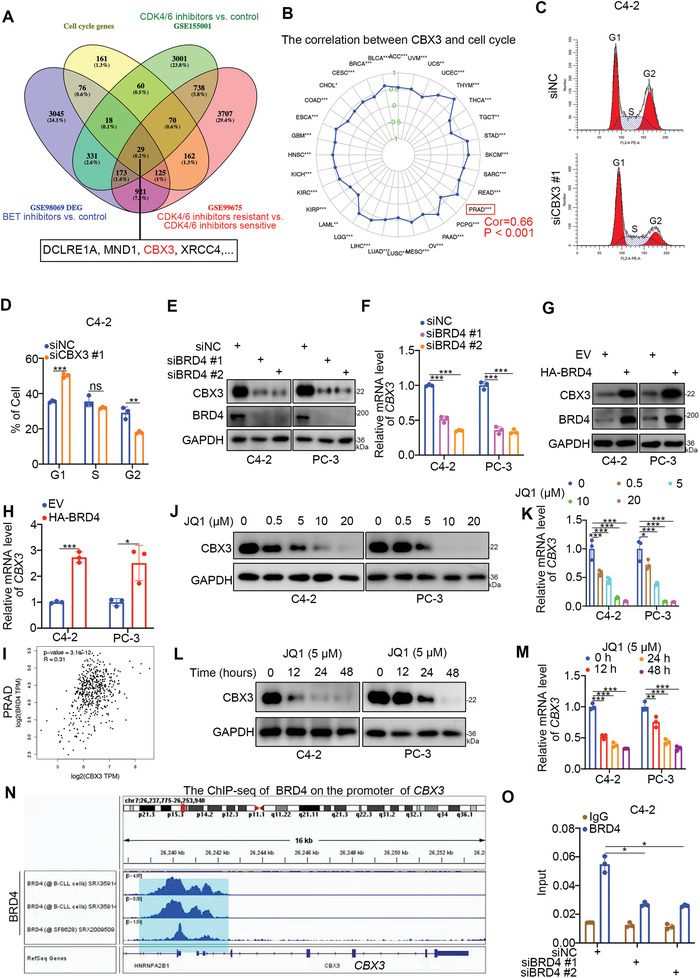
The cell cycle‐associated gene CBX3 is directly regulated by BRD4. A) The bioinformatic analysis the common genes associated with cell cycle, BET inhibitors related, CDK4/6 inhibitors related and CDK4/6 inhibitors resistance related. B) The TCGA sample data were divided into high and low groups for differential analysis using the median expression of the CBX3 gene, and the resulting differential genes were subsequently subjected to GSEA enrichment analysis to obtain the correlation between CBX3 and the cell cycle in multiple types of cancer. Correlation and P values as indicated. **, P < 0.01; ***, P < 0.001. PRAD, Prostate adenocarcinoma. C and D) C4‐2 cells were transfected with indicated siRNAs for 48 h. Cells were harvested for cell cycle analysis. Data presents as mean ± SEM with three replicates. Ns, not significant; **, P < 0.01; ***, P < 0.001. The cell cycle phases (G1, S, and G2) have been marked at the peaks. E—H) C4‐2 and PC‐3 cells were transfected with indicated siRNAs or plasmids for 48 h. Cells were harvested for Western blot analysis (E and G) and RT‐qPCR analysis (F and H). Data presents as mean ± SEM with three replicates. ***, P < 0.001. I, the correlation between CBX3 and BRD4 was analyzed by the GEPIA web tool (http://gepia.cancer‐pku.cn/). J and K) C4‐2 and PC‐3 cells were treated with a serial dose of JQ1 (0, 0.5, 5, 10, 20 µM) for 24 h. Cells were harvested for Western blot analysis (J) and RT‐qPCR analysis (K). Data presents as mean ± SEM with three replicates. *, P < 0.05; ***, P < 0.001. L and M) C4‐2 and PC‐3 cells were treated with 5 µM of JQ1. Cells were harvested at different time points and subjected to Western blot analysis (L) and RT‐qPCR analysis (M). Data presents as mean ± SEM with three replicates. **, P < 0.01; ***, P < 0.001. N) analysis of the ChIP‐seq of BRD4. O) C4‐2 cells were transfected with indicated siRNAs for 48 h. Cells were harvested for ChIP‐qPCR analysis. Data presents as mean ± SEM with three replicates. *, P < 0.05.

Given that cell cycle genes are closely related to the sensitivity of cancer cells to CDK4/6 inhibitors, we aimed to examine the candidates that influenced the anticancer effects of BET inhibitors and CDK4/6 inhibitors through a combination of RNA‐seq analysis of the treatment with CDK4/6 inhibitors versus control (GSE155001), – treatment with BET inhibitors versus control (GSE98069), CDK4/6 inhibitor‐resistant versus CDK4/6 inhibitor‐sensitive PC (GSE99675), and a cell cycle gene set (**Figure** **1**A). The data revealed that 29 genes were dysregulated after CDK4/6 inhibitor or BET inhibitors treatment and were associated with PC sensitivity to CDK4/6 inhibitors (Figure 1A). Interestingly, we found that CBX3, which we had previously studied,^[^ ^24^ ^]^ was one of the 29 genes (Figure 1A; Figure S1A,B, Supporting Information). It has been reported that CBX3 is involved in regulating the cell cycle of cancer cells.^[^ ^25^, ^26^, ^27^ ^]^ Here, we analyzed the relationship between CBX3 and the cell cycle and showed that CBX3 was almost positively correlated with the cell cycle in multiple types of malignancies (Figure 1B). In PC, the Spearman's correlation coefficient between CBX3 and the cell cycle was 0.66, and the P values was less than 0.001 (Figure 1B). Moreover, we showed that knockdown of CBX3 blocked the cell cycle in G1 phase in C4‐2 cells (Figure 1C,D; Figure S1C—E, Supporting Information), which confirmed the relationship between CBX3 and the cell cycle.

A previous study demonstrated that CBX3 was repressed by BET inhibitors (JQ1) and acted as a master regulator in CRPC.^[^ ^28^ ^]^ To exclude the possibility that the regulation of CBX3 by JQ1 was caused by off‐target effects, BRD4 was knocked down in both CRPC cell lines (Figure 1E,F). The downregulation of BRD4 decreased CBX3 levels in cells (Figure 1E,F). In contrast, overexpression of BRD4 increased CBX3 expression in C4‐2 and PC‐3 cells (Figure 1G,H; Figure S1F, Supporting Information). In addition to these findings, we demonstrated that there was a positive correlation between the mRNA levels of CBX3 and BRD4 in PC patient samples and other types of malignant tumors (Figure 1I; Figure S2A,B, Supporting Information). Consistent with a previous report,^[^ ^28^ ^]^ JQ1 decreased the protein and mRNA levels of CBX3 in PC cells (Figure 1J–M). Furthermore, ChIP‐seq analysis of BRD4 indicated that there was a binding peak of BRD4 on the promoter region of CBX3 (Figure 1N). ChIP‒qPCR analysis showed that BRD4 specifically bound to the promoter region of CBX3 in C4‐2 cells (Figure 1O), and JQ1 treatment decreased the binding of BRD4 to the promoter region of CBX3 (Figure S2C, Supporting Information). These results suggest that CBX3 is transcriptionally upregulated by BRD4 in PC cells.

### 2.2 CBX3 Regulates the Sensitivity of PC Cells to CDK4/6 Inhibitors

**Figure 2:**
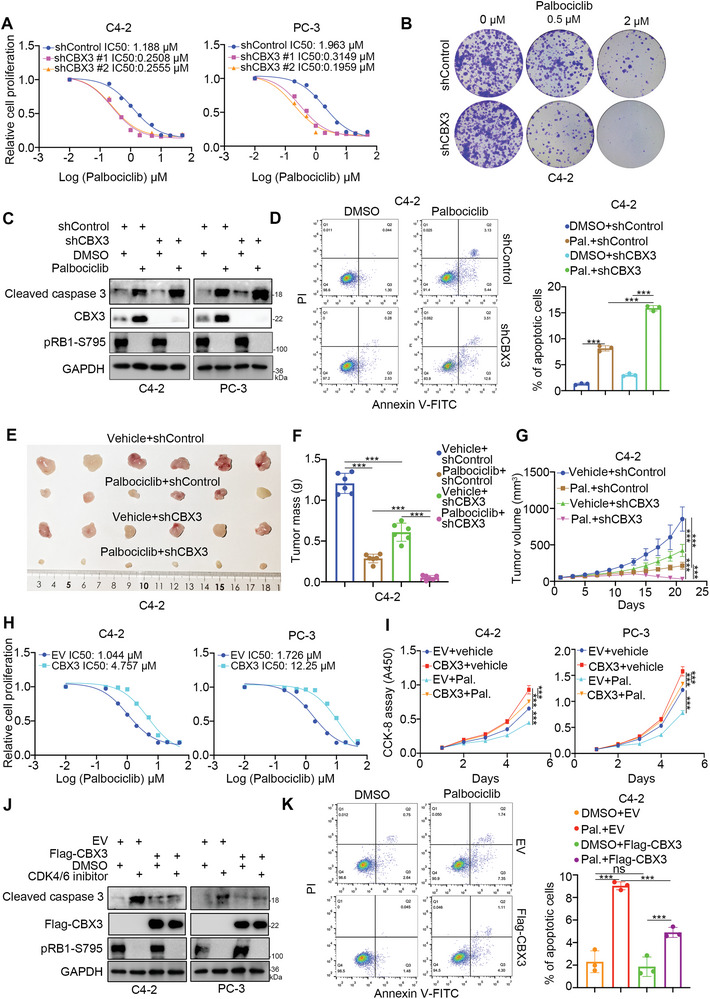
CBX3 regulates the sensitivity of PC cells to CDK4/6 inhibitors. A) C4‐2 and PC‐3 cells were transfected with indicated shRNAs for 72 h. Cells were treated with a serial dose of Palbociclib for 24 h and subjected to CCK8 assay. B) C4‐2 cells were transfected with indicated shRNAs for 72 h. Cells were treated with a serial dose (0, 0.5, 2 µM) of Palbociclib and subjected to colony formation assay. C and D) C4‐2 and PC‐3 cells were transfected with indicated shRNAs for 72 h. Cells were treated with or without 2 µM Palbociclib for 24 h. Cells were subjected to Western blot analysis (C) and FACS analysis (D). Data presents as mean ± SEM with three replicates. ***, P < 0.001. E—G) C4‐2 cells were transfected with indicated shRNAs for 72 h. After puromycin selection, cells were injected subcutaneously into the nude mice for xenograft assay. After the tumor volume ≈reached to 100 mm^3^, Palbociclib HCl (150 mg Kg^−1^) or Vehicle (Water) were orally administrated once every other day. The tumor image was shown in panel E, the tumor mass was shown in panel F, the growth curve was shown in panel E. Data presents as mean ± SEM with six replicates. ***, P < 0.001. H, C4‐2, and PC‐3 cells were transfected with indicated plasmids for 48 h. Cells were treated with a serial dose of Palbociclib for 24 h and subjected to CCK8 assay. I—K), C4‐2 and PC‐3 cells were transfected with indicated plasmids for 48 h. Cells were treated with or without 2 µM Palbociclib for 24 h. Cells were subjected to CCK‐8 assay (I), Western blot analysis (J) and FACS analysis (K). Data presents as mean ± SEM with three replicates. Ns, not significant; ***, P < 0.001.

CBX3 downregulation mainly blocks the G1 phase, which is similar to the effect of CDK4/6 inhibitors (palbociclib).^[^ ^15^ ^]^ The subsequent results showed that CBX3 knockdown increased the sensitivity of PC cells (C4‐2 and PC‐3) to CDK4/6 inhibitors (**Figure** **2**A,B; Figure S3A—C, Supporting Information). Then, we found that CBX3 depletion combined with palbociclib treatment resulted in more apoptotic C4‐2 and PC‐3 cells than palbociclib treatment alone (Figure 2C,D).The nude mouse xenograft assay showed that CBX3 inhibition enhanced the anticancer effect of palbociclib (Figure 2E–G). In contrast, CBX3 overexpression increased the IC50 values of palbociclib in C4‐2 and PC‐3 cells (Figure 2H; Figure S3D, Supporting Information). Similarly, we found that CBX3 overexpression decreased the anticancer effect of palbociclib on PC cells (Figure 2I–K). CBX3 was upregulated in the palbociclib treatment group and the Palbociclib‐resistant group (Figure S1A,B, Supporting Information). Thus, our data indicate that CBX3 modulates the sensitivity of PC cells to CDK4/6 inhibitors.

### 2.3 CBX3 Modulates CDK4/6 Inhibitor Sensitivity in an RB1‐Dependent Manner

**Figure 3:**
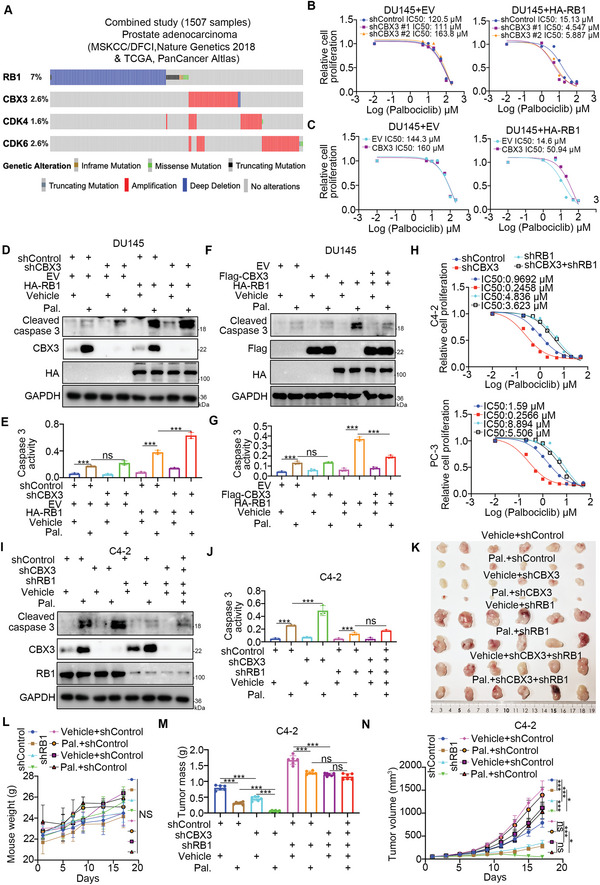
CBX3 modulates CDK4/6 inhibitor sensitivity in an RB1‐dependent manner. A) analysis of TCGA‐PRAD dataset. B and C) DU145 cells were transfected with indicated shRNAs for plasmids for 72 h. Cells were with a serial dose of Palbociclib for 24 h and subjected to CCK8 assay. D—G) DU145 cells were transfected with indicated shRNAs for plasmids for 72 h. Cells were treated with or without 20 µM Palbociclib for 24 h. Cells were subjected to Western blot analysis (D and F) and caspase 3 activity assay (E and G). Data presents as mean ± SEM with three replicates. Ns, not significant; ***, P < 0.001. H) C4‐2 and PC‐3 cells were transfected with indicated shRNAs for 72 h. Cells were treated with a serial dose of Palbociclib for 24 h and subjected to CCK8 assay. I and J) C4‐2 cells were transfected with indicated shRNAs for 72 h. Cells were treated with or without 2 µM Palbociclib for 24 h. Cells were subjected to Western blot analysis and caspase 3 activity assay. Data presents as mean ± SEM with three replicates. Ns, not significant; ***, P < 0.001. K—N) C4‐2 cells were transfected with indicated shRNAs for 72 h. After puromycin selection, cells were injected subcutaneously into the nude mice for xenograft assay. After the tumor volume ≈reached to 100 mm^3^, Palbociclib HCl (150 mg Kg^−1^) or Vehicle (Water) were orally administrated once every other day. The tumor image was shown in panel K, the mouse weight was shown in panel (L), the tumor mass was shown in panel (M), the growth curve was shown in panel (N). Data presents as mean ± SEM with six replicates. ***, P < 0.001.

Since CDK4/6 inhibitors blocked cell cycle progression mainly by downregulating the CDK4/6‐mediated phosphorylation of RB1,^[^ ^29^ ^]^ we analyzed the publicly available second‐generation sequencing data of PC patient specimens and found that CBX3 overexpression was mutually exclusive with RB1 deletion (**Figure** **3**A). It has been well documented that the expression level of RB1 plays an important role in determining the sensitivity of cancer cells to CDK4/6 inhibitors.^[^ ^29^ ^]^ Our data demonstrated that the IC50 values of palbociclib in RB1‐deficient DU145 cells were much higher than those of in RB‐proficient PC cells, such as LNCaP, 22Rv1, C4‐2 and PC‐3 cells (Figure S3E, Supporting Information). These data indicated that CBX3 modulated the sensitivity of PC cells to palbociclib. Given that the expression level of CBX3 in 22Rv1 cells was higher than the expression level of CBX3 in C4‐2 and LNCaP cells, the IC50 values of palbociclib in 22Rv1 cells were slightly higher than those in C4‐2 and LNCaP cells (Figure S3E—G, Supporting Information). Interestingly, our results showed that the expression level of CBX3 in LNCaP and C4‐2 cells was higher than that in PC‐3 cells, and the expression level of RB1 in PC‐3 cells lower than that in LNCaP and C4‐2 cells,^[^ ^22^, ^30^ ^]^ which might explain why the IC50 values of palbociclib in PC‐3 cells were slightly higher than those in LNCaP or C4‐2 cells (Figure S3E—G, Supporting Information). We further explored the relationship between CBX3 and RB1 in regulating the sensitivity to Palbociclib and showed that knockdown or overexpression of CBX3 had no effect on the IC50 values of palbociclib in RB1‐deficient DU145 cells (Figure 3B,C; Figure S4A, Supporting Information). Moreover, rescuing RB1 expression in DU145 cells restored the regulatory effect of CBX3 on palbociclib sensitivity (Figure 3B–G). In contrast, knockdown of RB1 in C4‐2 and PC‐3 cells decreased the regulatory effect of CBX3 on palbociclib sensitivity of in vitro and in vivo (Figure 3H–K). Thus, our results suggest that RB1 is indispensable for CBX3‐mediated regulation of the sensitivity of PC cells to CDK4/6 inhibitors.

### 2.4 CBX3 Interacts with RB1 to Release E2F1 in PC Cells

**Figure 4:**
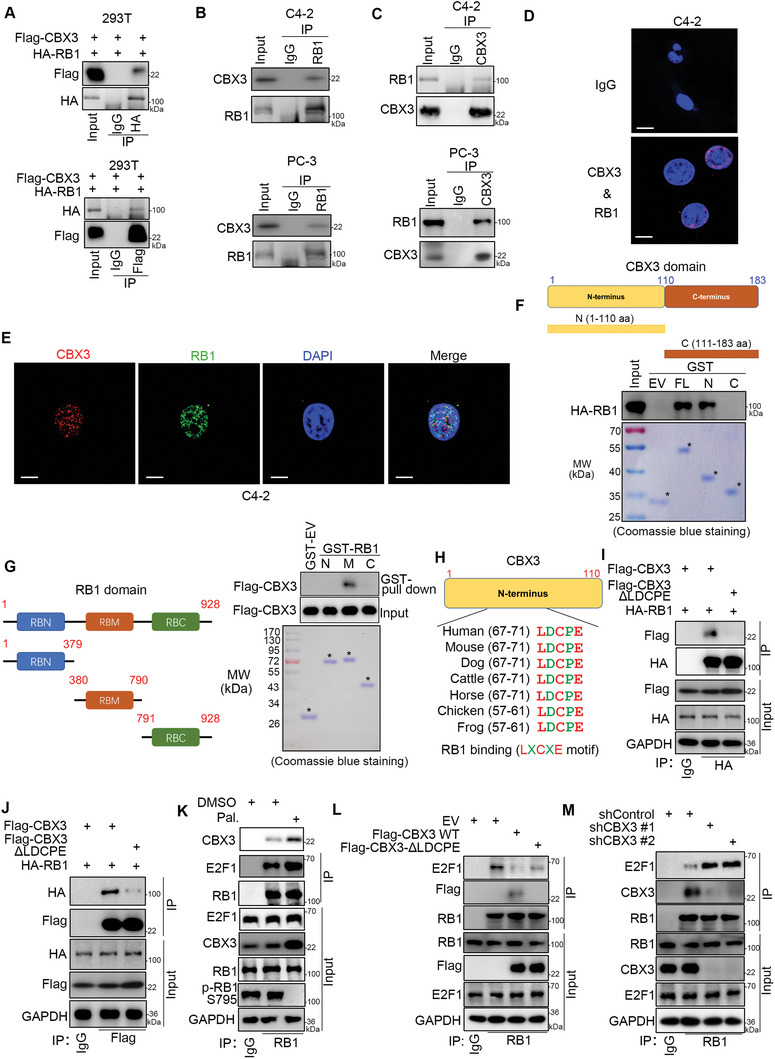
CBX3 interacts with RB1 to release E2F1 in PC cells. A) the whole cell lysate (WCL) of 293T cells after transfected with indicated plasmids were harvested for immunoprecipitation (IP) assay. B and C) the WCL of C4‐2 and PC‐3 cells were harvested for IP assay. D) the PLA assay was performed in C4‐2 cells by using the indicated antibodies. E) the immunofluorescence assay was performed in C4‐2 cells by using the indicated antibodies. F) schematic diagram depicting the domain of CBX3. GST‐pulled down assay was performed by using the recombinant protein of CBX3. G) schematic diagram depicting the domain of RB1. GST‐pulled down assay was performed by using the recombinant protein of RB1. H) schematic diagram depicting the “LXCXE” motif in CBX3. I and J) C4‐2 cells were transfected with indicated plasmids for 48 h. Cells were harvested for IP assay. K) C4‐2 cells were treated with or without 1 µM Palbociclib for 24 h. Cells were harvested for IP assay. L and M) C4‐2 cells were transfected with indicated plasmids or shRNAs for 48 h. Cells were harvested for IP assay.

To investigate the relationship between CBX3 and RB1, we overexpressed Flag‐tagged CBX3 and HA‐tagged RB1 in 293T cells (**Figure** **4**A). The immunoprecipitation assay demonstrated that ectopically overexpressed CBX3 and RB1 interacted with each other in 293T cells (Figure 4A). Then, we demonstrated that endogenously expressed CBX3 reciprocally interacted with RB1 in C4‐2 and PC‐3 cells (Figure 4B,C). Moreover, the proximity ligation assay (PLA) showed that CBX3 bound to RB1 in C4‐2 cells (Figure 4D; Figure S4A, Supporting Information), and we could not observe a PLA signal of CBX3 and RB1 in RB1‐deficient DU145 cells, which indicated that the PLA signal of CBX3 and RB1 was specific (Figure S4B, Supporting Information). Then, the immunofluorescence assay indicated that CBX3 colocalized with RB1 in the nucleus of C4‐2 cells (Figure 4E). To further determine which region(s) in CBX3 contributes to its interaction with RB1, we constructed two GST‐tagged CBX3 recombinant proteins (Figure 4F). The GST pull‐down assay indicated that the full length and N‐terminus (111‐183 aa) but not the C‐terminus (1‐110 aa) of CBX3 bound to RB1 (Figure 4F). In addition, we constructed three GST‐tagged RB1 recombinant proteins (Figure 4G). We showed that the RB1‐M recombinant protein (380—790 aa) mediated the interaction between RB1 and CBX3 by GST pull‐down assays (Figure 4G). It has been reported that RB1 interacts with E2F1 through its M domain by recognizing the “LXCXE” motif of E2F1.^[^ ^31^ ^]^ We found that there was a conserved “LXCXE” motif in the N‐terminus of CBX3 (Figure 4H). We constructed a mutant (Flag‐CBX3 delta LDCPE) of CBX3 that lacked the “LXCXE” motif (Figure 4H,I). The co‐IP assay demonstrated that the absence of the “LDCPE” sequence in CBX3 resulted in decreased the binding between CBX3 and RB1 in C4‐2 cells (Figure 4I,J). In addition, CDK4/6 inhibitor treatment has been reported to strengthen the binding between RB1 and E2F1 by decreasing the phosphorylation of RB1.^[^ ^32^ ^]^ Similarly, we showed that treatment with palbociclib enhanced the interaction of RB1 and E2F1 and the interaction of RB1 and CBX3 in C4‐2 cells (Figure 4K). In addition, we also demonstrated that the nonphosphorylatable RB1 mutant—RB1 delta CDK (RB1 ΔCDK), in which fifteen major CDK phosphorylation sites are mutated to alanine,^[^ ^33^ ^]^ bound more CBX3 or E2F1 than the wild‐type (WT) RB1 construct in C4‐2 cells (Figure S4C, Supporting Information). Given that RB1 bound to E2F1 to inhibit the function of E2F1 in cells, we examined whether CBX3 could release E2F1 from RB1. We revealed that overexpression of CBX3 WT but not the CBX3 delta LDPCE mutant (CBX3 ΔLDPCE) decreased RB1 binding to E2F1 in C4‐2 cells (Figure 4L; Figure S4D, Supporting Information). Moreover, we demonstrated that knockdown of CBX3 increased the amount of E2F1 bound to RB1 in C4‐2 cells (Figure 4M). In addition, we demonstrated that depleting of CBX3 affected the RB1‐E2F1 interaction in the presence of palbociclib (Figure S4E, Supporting Information). We also showed that overexpression of CBX3 WT but not CBX3 delta LDPCE mutant affected the RB1‐E2F1 interaction in the presence of palbociclib (Figure S4F, Supporting Information). Overexpression of CBX3 WT but not CBX3 delta LDPCE mutant rescued the effect of CBX3 knockdown on the interaction of RB1 and E2F1 in C4‐2 cells (Figure S4G, Supporting Information). Thus, our results suggest that CBX3 binds with the M domain of RB1 to release E2F1 in PC cells.

### 2.5 CBX3 Regulates Cell Cycle‐Related Genes through The RB1/E2F1 Axis

**Figure 5:**
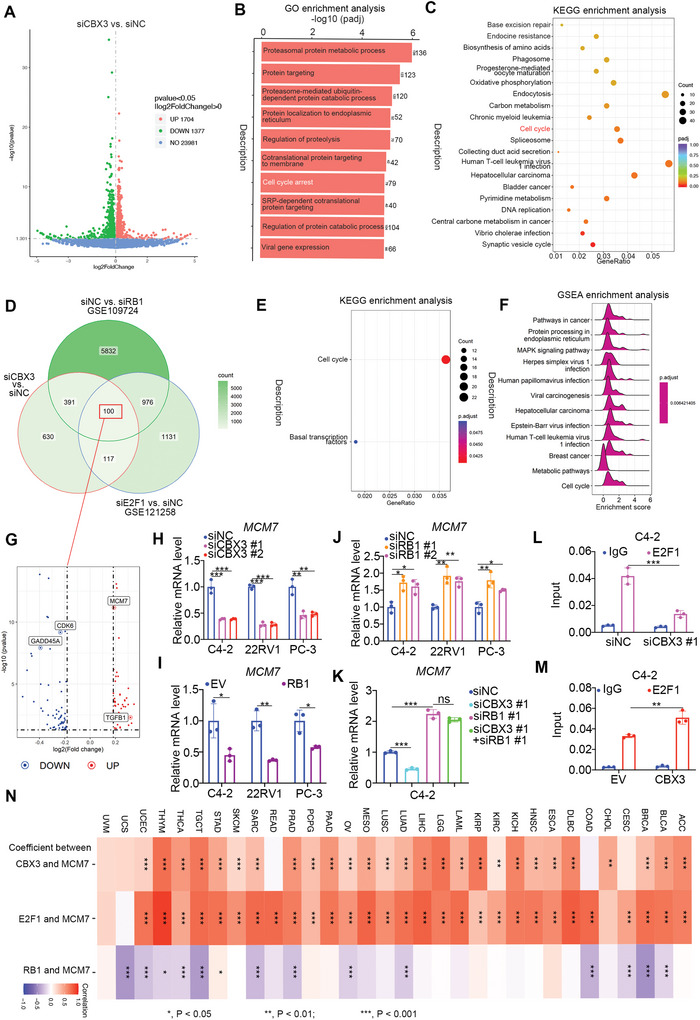
CBX3 regulates cell cycle‐related genes through the RB1/E2F1 axis. A—C) C4‐2 cells were transfected with indicated siRNAs for 48 h. Cells were harvested for RNA‐seq analysis (A). And subsequent GO (B) and KEGG (C) enrichment analysis were performed. D—G) analysis of the RNA‐seq of knockdown of CBX3 in C4‐2 cells, GSE109724, and GSE121258 to found the common regulated genes (D). KEGG and GSEA analysis was performed. H—J) C4‐2, 22RV1, and PC‐3 cells were transfected with indicated siRNAs or plasmids for 48 h. Cells were harvested for RT‐qPCR assay. Data presents as mean ± SEM with three replicates. *, P < 0.05; **, P < 0.01; ***, P < 0.001. K) C4‐2 cells were transfected with indicated siRNAs for 48 h. Cells were harvested for RT‐qPCR assay. Data presents as mean ± SEM with three replicates. Ns, not significant; ***, P < 0.001. L and M) C4‐2 cells were transfected with indicated siRNAs or plasmids for 48 h. Cells were subjected to ChIP‐qPCR assay by using the indicated antibodies. Data presents as mean ± SEM with three replicates. **, P < 0.01; ***, P < 0.001. N) analysis of TCGA dataset to showed the correlation between CBX3 and MCM7, E2F1 and MCM7, and RB1 and MCM7. P values as indicated.

To further explore the relationship between CBX3 and the RB1/E2F1 axis, we performed RNA‐seq analysis of C4‐2 cells after CBX3 knockdown (**Figure** **5**A). Gene Ontology (GO) enrichment analysis and Kyoto Encyclopedia of Genes and Genomes (KEGG) enrichment analysis demonstrated that CBX3 was closely associated with the cell cycle (Figure 5B,C). Then, we analyzed the RNA‐seq results of CBX3, RB1 (GSE109724), and E2F1 (GSE121258) (Figure 5D). We showed that 100 genes were simultaneously regulated by CBX3, RB1 and E2F1 (Figure 5D). We also demonstrated that these genes were associated with the cell cycle by KEGG and gene set enrichment analysis (GSEA) (Figure 5E,F). Among these genes, the cell cycle‐related gene, MCM7, is reported to be upregulated by E2F1.^[^ ^34^ ^]^ Replication initiation at the G1/S phase transition activates mini‐chromosome maintenance (MCM) helicases, which allows DNA unwinding.^[^ ^38^ ^]^ MCM7 is a member of the MCM complex and is responsible for DNA replication.^[^ ^39^ ^]^ A study showed that ≈50% of the PC specimens studied exhibited MCM7 gene amplification and that 60% of the aggressive PC specimens showed elevated protein expression of MCM7.^[^ ^39^ ^]^ Significant associations between the amplification or overexpression of MCM7 and relapse, local invasion, and a high tumor grade were observed.^[^ ^39^ ^]^ Our data showed that MCM7 expression was increased by CBX3 and suppressed by RB1 in PC cells (Figure 5G–I; Figure S5A, Supporting Information). In addition to MCM7, we showed that other RB1‐E2F1‐regulated genes (such as, TGFB1, CDK6, GADD45A) were affected by CBX3 and RB1 in PC cells (Figure 5G; Figure S5B—D, Supporting Information). Furthermore, we demonstrated that RB1 knockdown attenuated the regulatory effect of CBX3 on the expression of MCM7 in RB1‐proficient C4‐2 cells but not in the RB1‐deficient DU145 cells (Figure 5K; Figure S5E,F, Supporting Information). The ChIP‐atlas dataset, we demonstrated that there were common CBX3, E2F1 and RB1 binding regions in the promoter of CBX3 (Figure S4G, Supporting Information). ChIP‒qPCR analysis indicated that CBX3 knockdown decreased the binding of E2F1 to the promoter of MCM7 (Figure 5L). Moreover, C4‐2 cells were transfected with GV592‐EV or GV592‐MCM7 plasmids, and a luciferase reporter assay demonstrated that ectopically overexpressed E2F1 increased the luciferase activity of the GV592‐MCM7 promoter, while cotransfection of CBX3 decreased these effects (Figure S5H,I, Supporting Information). In contrast, CBX3 overexpression promoted E2F1 binding to the promoter of MCM7 in C4‐2 cells (Figure 5M). Overexpression CBX3 WT but not CBX3 delta LDCPE mutant increased the luciferase activity of the GV592‐MCM7 promoter after the overexpressed E2F1 with or without palbociclib treatment (Figure S5J,K, Supporting Information). In addition, we also showed that overexpression CBX3 WT but not CBX3 delta LDCPE mutant led more E2F1 binding to the promoter of MCM7 in C4‐2 cells with or without palbociclib treatment (Figure S5L, Supporting Information). Furthermore, analysis of pancancer TCGA data indicated that the expression of CBX3 and E2F1 was almost positively correlated with that of MCM7, but the expression of RB1 was negatively correlated with the expression of MCM7 in multiple types of malignancies (Figure 5N; Figure S5M, Supporting Information). Taken together, these data indicate that CBX3 modulates cell cycle‐related genes through RB1/E2F1 in PC.

### 2.6 PLK1 Phosphorylates CBX3 to Promote CBX3 Binding to RB1 in PC

**Figure 6:**
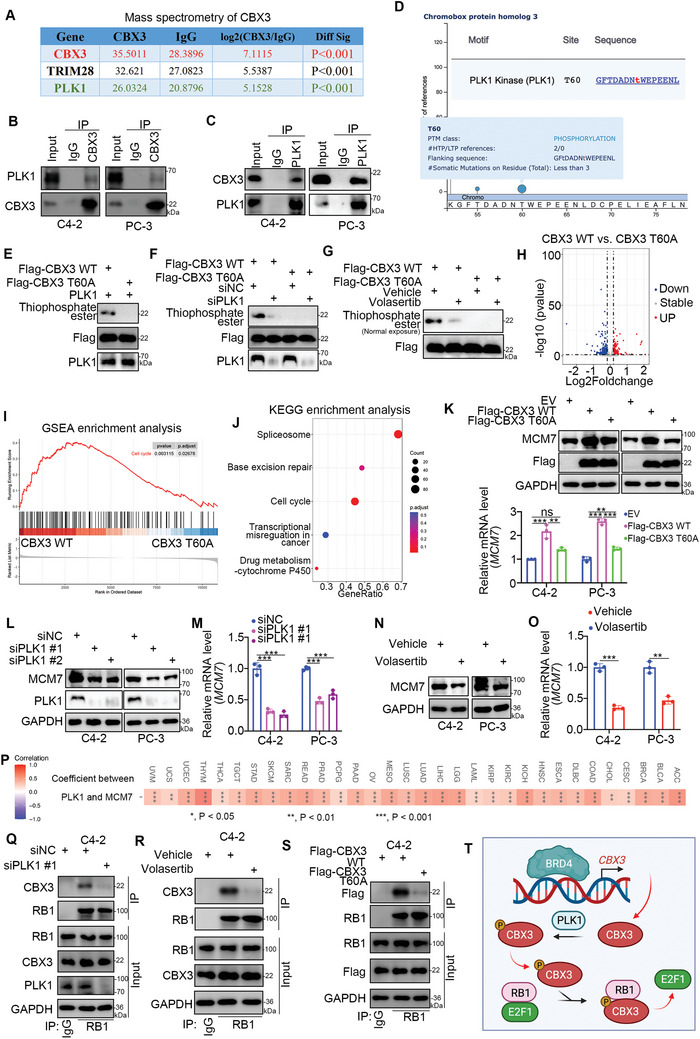
PLK1 phosphorylates CBX3 to promote CBX3 binding to RB1 in PC. A) analysis of mass spectrometry of CBX3. B and C) C4‐2 and PC‐3 cells were harvested for IP assay. D) analysis of the Scansite web tool (https://scansite4.mit.edu/#home) and PhosphoSitePlus web tool (https://www.phosphosite.org/). E) the CBX3 WT and CBX3 T60A proteins were translated in vitro. These proteins were purified by Protein G agarose and primary antibodies (Flag‐tag antibody). Then, the proteins were incubated with PLK1 and ATP‐γ‐S and subjected to Western blot analysis. F and G) C4‐2 cells were transfected with indicates constructs or reagents for 48 h. Cells were harvested for IP assay and Western blot analysis. H—J) C4‐2 cells were transfected with indicates plasmids for 48 h. Cells were subjected to RNA‐seq analysis. K–M) C4‐2 and PC‐3 cells were transfected with indicates plasmids or siRNAs for 48 h. Cells were subjected to Western blot analysis and RT‐qPCR analysis. Data presents as mean ± SEM with three replicates. ns, not significant; ***, P < 0.001. N and O) C4‐2 and PC‐3 cells were treated with or without 1 µM volasertib for 24 h. Cells were subjected to Western blot analysis and RT‐qPCR assay. Data presents as mean ± SEM with three replicates. **, P < 0.01; ***, P < 0.001. P) analysis of TCGA dataset to showed the correlation between PLK1 and MCM. P values as indicated. Q—S) C4‐2 cells were transfected with indicated constructs or treated with indicated reagent for 48 h. Cells were harvested for IP assay. T) a model depicting that BRD4 transcriptionally increases the expression of CBX3, the up‐regulated CBX3 was phosphorylated by the PLK1 and bound with RB1 to release E2F1.

The above data indicated that CBX3 was an ideal candidate for enhancing the anticancer activity of palbociclib. However, no small molecule inhibitor targeting CBX3 has been identified to date. We reanalyzed the previously published mass spectrometry data on CBX3 binding partners^[^ ^20^ ^]^ and found that PLK1 could be the binding partner of CBX3 (**Figure** **6**A). The reciprocal co‐IP assay indicated that endogenously expressed PLK1 interacted with CBX3 in C4‐2 and PC‐3 cells (Figure 6B,C). The Scansite web tool (https://scansite4.mit.edu/#home) and PhosphoSitePlus web tool (https://www.phosphosite.org/) predicted that threonine 60 (T60) of CBX3 could be phosphorylated by PLK1 (Figure 6D). Then, we constructed the CBX3 T60A mutant to mimic dephosphorylated CBX3. The kinase assay showed that PLK1 mediated the phosphorylation of CBX3 at T60 (Figure 6E–G).

To explore the relationship between PLK1 and CBX3, we performed RNA‐seq analysis of C4‐2 cells after the overexpression of CBX3 WT or CBX3 T60A constructs (Figure 6H; Figure S6A,B, Supporting Information). GSEA and KEGG enrichment analysis indicated that compared to CBX3 T60A overexpression, CBX3 WT overexpression activated the cell cycle signaling pathway in C4‐2 cells (Figure 6I,J). Similarly, we demonstrated that CBX3 T60A overexpression did not greatly increase the expression of MCM7 compared with that in C4‐2 and PC‐3 cells with CBX3 WT overexpression (Figure 6K). Furthermore, our data showed that knockdown of PLK1 or treatment with a PLK1 inhibitor (volasertib) decreased the expression of MCM7 in C4‐2 and PC‐3 cells (Figure 6L–O). Moreover, TCGA dataset analysis showed that the expression of PLK1 was positively correlated with that of MCM7 (Figure 6P). These data indicated that PLK1‐mediated phosphorylation of CBX3 at T60 regulated the cell cycle‐related effect of CBX3. Notably, we found that the CBX3 T60 phosphorylation site was adjacent to the “LXCXE” motif of CBX3 (Figure 4F). Subsequent analysis showed that PLK1 inhibition by siRNAs or a specific inhibitor (volasertib) decreased the binding of CBX3 and RB1 in C4‐2 cells (Figure 6Q,R). The CBX3 T60A mutant bound less RB1 than CBX3 WT in C4‐2 cells (Figure 6S). Furthermore, the in vitro PLK1 kinase assay was performed by inoculating the GST‐WT CBX3 or GST‐CBX3 T60A mutant with or without PLK1, and the in vitro protein binding assay was used to transcribe and translate RB1 in vitro to investigate whether the RB1‐CBX3 interaction was dependent on phosphorylation (Figure S6C, Supporting Information). We showed that T60 phosphorylation of CBX3 enhanced the binding of CBX3 and RB1, but no such effect was observed in the CBX3‐T60A mutant (Figure S6C, Supporting Information). Given that CBX3 could release E2F1 from RB1, we demonstrated that volasertib treatment with or without palbociclib treatment decreased the binding of CBX3 and RB1 and increased the interaction between E2F1 and RB1 in C4‐2 cells (Figure S6D, Supporting Information). To further explore whether PLK1 activated E2F1 through CBX3 phosphorylation, we showed that CBX3 knockdown in PLK1‐overexpressing C4‐2 cells increased the binding between RB1 and E2F1 and decreased target gene (MCM7) expression (Figure S6E,F, Supporting Information). Overexpressing CBX3 WT after CBX3 knockdown to rescue CBX3 expression decreased the binding between E2F1 and RB1 and promoted target gene expression (Figure S6E,F, Supporting Information). In addition, this effect was not obvious after overexpression of the CBX3 T60A mutant (Figure S6E,F, Supporting Information). Moreover, a luciferase reporter assay demonstrated that overexpression of CBX3 WT but not the CBX3 T60A mutant increased the luciferase activity of the GV592‐MCM7 promoter after the overexpression of E2F1 in C4‐2 cells (Figure S6G, Supporting Information). Moreover, our results demonstrate that overexpression of the dephosphorylated CBX3 T60A mutant enhanced the sensitivity of PC cell lines to palbociclib less than the overexpression of CBX3 WT (Figure S6H, Supporting Information). Therefore, these data suggest that PLK1 phosphorylates CBX3 to enhance the binding between CBX3 and RB1 and release E2F1 from RB1 in PC cells (Figure 6T).

### 2.7 A Dual BRD4/PLK1 Inhibitor Sensitizes PC Cells to the Anticancer Effects of the CDK4/6 Inhibitor Partially through CBX3

**Figure 7:**
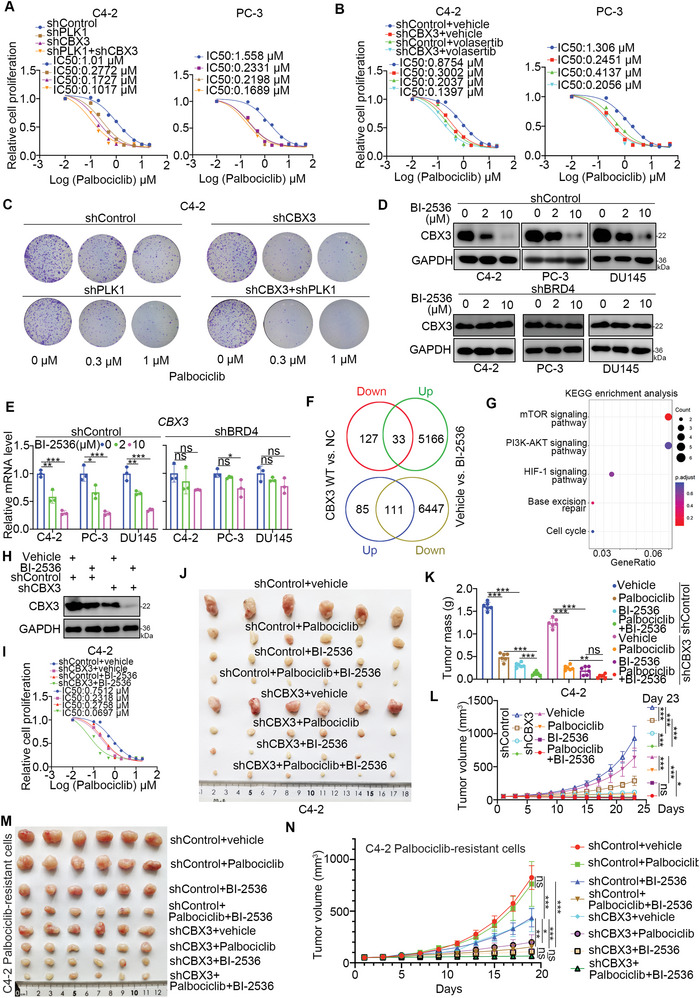
A dual BRD4/PLK1 inhibitor sensitizes PC cells to the anticancer effects of the CDK4/6 inhibitor partially through CBX3. A) C4‐2 and PC‐3 cells were transfected with indicated shRNAs for 72 h. Cells were treated with a serial dose of Palbociclib for 24 h and subjected to CCK8 assay. B) C4‐2 and PC‐3 cells were transfected with indicated shRNAs for 48 h. Then, these cells were treated with or without 1 µM volasertib for 24 h. Cells were treated with a serial dose of Palbociclib for 24 h and subjected to CCK8 assay. C) C4‐2 cells were transfected with indicated shRNAs for 72 h. Cells were treated with a serial dose of Palbociclib and subjected to colony formation assay. D and E) C4‐2, PC‐3, and DU145 cells were transfected with indicated shRNAs for 48 h. Then, these cells were treated with a serial dose (0, 2, 10 µM) of BI‐2536 for 24 h and subjected to Western blot analysis and RT‐qPCR assay. Data presents as mean ± SEM with three replicates. Ns, not significant; *, P < 0.05; **, P < 0.01; ***, P < 0.001. F and G) C4‐2 cells were treated with or without 10 µM BI‐2536 for 24 h. Cells were subjected to RNA‐seq analysis. Analysis of co‐regulated genes of CBX3 and BI‐2536 treatment. H and I) C4‐2 cells were transfected with indicated shRNAs for 48 h. Then, these cells were treated with or without 10 µM BI‐2536 for 24 h. Cells were harvested for Western blot analysis and CCK‐8 assay. J—L) C4‐2 cells were transfected with indicated shRNAs for 72 h. After puromycin selection, cells were injected subcutaneously into the nude mice for xenograft assay. After the tumor volume approximately reached to 100 mm3, Palbociclib HCl (150 mg Kg^−1^) or Vehicle (Water) were administrated orally once every other day, Vehicle or BI‐2535 (50 mg Kg^−1^) were intraperitoneal injected once every other three day. The tumor image was shown in panel J, the tumor mass was shown in panel K, the growth curve was shown in panel L. Data presents as mean ± SEM with six replicates. *, P < 0.05; ***, P < 0.001. M and N) C4‐2 Palbociclib‐resistance cells were transfected with indicated shRNAs for 72 h. After puromycin selection, cells were injected subcutaneously into the nude mice for xenograft assay. After the tumor volume ≈reached to 100 mm3, Palbociclib HCl (150 mg Kg^−1^) or Vehicle (Water) were administrated orally once every other day, Vehicle or BI‐2535 (50 mg Kg^−1^) were intraperitoneal injected once every other three day. The tumor image was shown in panel (M), the growth curve was shown in panel (N). Data presents as mean ± SEM with six replicates. Ns, not significant; *, P < 0.05; **, P < 0.01; ***, P < 0.001.

Since PLK1 modulates the interaction between CBX3 and RB1, we examined whether PLK1‐CBX3 regulated the sensitivity of PC to CDK4/6 inhibitors. First, we showed that inhibiting of PLK1 decreased the IC50 values of palbociclib (**Figure** **7**A; Figure S7A, Supporting Information). Knockdown of PLK1 and CBX3 slightly decreased the IC50 values of palbociclib compared with knockdown of PLK1 or CBX3 alone in PC cells (Figure 7A; Figure S7A, Supporting Information). Then, we showed that treatment with volasertib in the CBX3 knockdown group slightly decreased the IC50 values of Palbociclib compared to knockdown of CBX3 alone in C4‐2 and PC‐3 cells (Figure 7B). The in vitro cell proliferation assay also indicated that depletion of PLK1 expression or inhibition of PLK1 function enhanced the anticancer effect of palbociclib in prostate cancer cells (Figure 7C; Figure S7B, Supporting Information). This phenomenon was diminished after co‐knockdown of CBX3 (Figure 7C; Figure S7B, Supporting Information). These results indicated that CBX3 mediates the regulation of the sensitivity of PC cells to palbociclib via PLK1.

The above study showed that the expression level of CBX3 was regulated by BRD4 and that the phosphorylation of CBX3 was modulated by PLK1. A BRD4/PLK1 dual inhibitor (BI‐2536) has been developed.^[^ ^35^ ^]^ We were curious about whether BI‐2536 promotes the anticancer efficiency of CDK4/6 inhibitors in prostate cancer. First, we showed that BI‐2536 treatment decreased the expression of CBX3, and these phenomena were not obvious after knockdown of BRD4 in C4‐2, PC‐3, and DU145 cells (Figure 7D,E; Figure S7C, Supporting Information). In addition, C4‐2 cells were treated with BI‐2536 and subjected to transcriptome analysis (Figure S7D, Supporting Information). KEGG enrichment analysis indicated that treatment with BI‐2536 inactivated multiple signaling pathways, including the cell cycle, PI3K‐AKT signaling pathway, Ras signaling pathway, and prostate cancer‐related pathways (Figure S7E, Supporting Information). Then, we revealed that 33 plus 111 genes could be regulated by both CBX3 and BI‐2536 treatment (Figure 7F). The subsequent KEGG enrichment of these 33 plus 111 genes indicated that several signaling pathways, such as the mTOR signaling pathway, PI3K‐AKT signaling pathway, HIF‐1 signaling pathway, base excision repair and cell cycle‐related pathways, were involved (Figure 7G). Furthermore, the in vitro and in vivo cell proliferation assays revealed that BI‐2536 treatment enhanced the anticancer effect of palbociclib, which was attenuated after depletion of CBX3 in prostate cancer cells or palbociclib‐resistant C4‐2 cells (Figure 7H–N). In addition, we also showed that rescue the expression of CBX3 decreased the effect of BI‐2536 on improving the sensitivity of C4‐2 cells to palbociclib (Figure S7F,G, Supporting Information). Together, our data suggest that the expression level of CBX3 partially determines the anticancer effect of the dual BRD4/PLK1 inhibitor combined with the CDK4/6 inhibitor on PC (Figure S7H, Supporting Information).

## 3 Discussion

In this study, we found that CBX3 acted as a bridge to connect the therapeutic effects of BET and CDK4/6 inhibitors on PC. We showed that the cell cycle‐related gene CBX3 was directly downregulated by BET inhibitors and that CBX3 regulated the sensitivity of PC cells to CDK4/6 inhibitors in an RB1/E2F1‐dependent manner. Although there was still a weak but firm upregulation of CBX3 in palbociclib‐sensitive PC cells compared to those of in palbociclib‐resistant group (Figure S1B, Supporting Information), the nude mice xenograft study showed that knockdown of CBX3 also overcome the resistance of palbociclib in C4‐2 palbociclib‐resistant cells (Figure 7M–O). Thus, we believed that CBX3 plays a role in determining the sensitivity of palbociclib in PC cells. CBX3 is a component of heterochromatin‐associated proteins that binds specifically to trimethylated lysine 9 of histone H3 (H3K9me3), leading to the formation of heterochromatin.^[^ ^20^ ^]^ CBX3 modulates numerous cellular processes, such as cell growth, cell differentiation, and DNA damage, by suppressing the expression of various genes.^[^ ^20^ ^]^ CBX3 has recently been identified as a potential marker of carcinogenesis due to its high expression in several human cancer types, including colorectal cancer, lung cancer, hepatocellular carcinoma, and pancreatic cancer. This finding suggests that CBX3 play a significant role in promoting the development of cancer cells.^[^ ^20^, ^24^, ^36^ ^]^ Aberrant CBX3 expression contributes to the progression of various cancer types through the epigenetic regulation of cancer development and growth‐related genes.^[^ ^27^, ^37^ ^]^ In PC, CBX3 regulates AR signal transduction activity and promotes c‐MYC expression, thereby contributing to the progression of tumor growth.^[^ ^37^ ^]^ Notably, we directly demonstrated that BRD4 transcriptionally increased the expression of CBX3 in CRPC cells. Additionally, CBX3 is an AR‐independent master associated with BET inhibitor treatment of CRPC.^[^ ^28^ ^]^ Here, we found that the CDK4/6 inhibitor treatment increased the expression of CBX3 (Figures S1A and S2C, Supporting Information), which was also upregulated in the CDK4/6 inhibitor‐resistant cells (Figure S1B, Supporting Information). In addition, we noticed that RB1 overexpression or depletion had little effect on the protein levels of CBX3 (Figure 3D,I), which indicated that the mechanism by which palbociclib regulates the expression of CBX3 needs to be further studied.

We noticed that CBX3 promoted the G1‐S cell cycle transition in CRPC cells. In tongue squamous cell carcinoma, analogous results were observed, and CBX3 overexpression inhibited p21 expression and promoted the G1/S phase transition in the cell cycle.^[^ ^26^ ^]^ Furthermore, CBX3 was reported to inhibit p21 in a CDK6‐independent manner to promote cell cycle progression in colon cancer.^[^ ^38^ ^]^ We examined the effect of palbociclib on tumor cells and showed that after CBX3 knockdown or overexpression in Rb1‐deficient prostate cancer cells, RB1 was found to be a crucial component in CBX3‐mediated regulation of PC cell sensitivity to CDK4/6 inhibitors. Mechanistically, CBX3 binds to the pocket domain of RB1 to release E2F1 in PC cells. Bioinformatics analysis after CBX3 knockdown showed that CBX3 was closely related to the cell cycle. After analyzing datasets related to CBX3, RB1 and E2F1, we found that CBX3 regulated PC cell cycle‐related genes through Rb1/E2F1. Here, we demonstrated that CBX3 interacted with RB1 to release E2F1 and promoted cell cycle progression. However, Patima et al. showed that RB1/p130 recruited CBX3 to suppress genes that promote proliferation in adult cardiac myocytes,^[^ ^39^ ^]^ which contradicts our findings. We hypothesize that the effect of the CBX3/RB1 axis on cells might be organ‐ or tissue‐specific.

CBX3 is an ideal candidate for enhancing the anticancer activity of palbociclib; however, no small molecule inhibitors of CBX3 have been found thus far. According to the Scansite website, it is predicted that threonine 60 of CBX3 may be phosphorylated by PLK1. Through experiments, we found that phosphorylation of CBX3 by PLK1 promotes the binding of CBX3 and RB1 in PC. PLK1 is a serine/threonine protein kinase with multiple functions that is involved in DNA replication, chromosome segregation, and various stress response processes.^[^ ^40^ ^]^ PLK1 is a key cell cycle regulatory factor that functions through chromosome separation.^[^ ^41^ ^]^ Inhibition PLK1 leads to the stagnation of cells in the mitotic phase.^[^ ^42^ ^]^ Studies have shown that PLK1 is implicated in resistance to several chemotherapy drugs used to treat cancer, such as doxorubicin, paclitaxel, metformin, and gemcitabine.^[^ ^41^ ^]^ Drugs targeting PLK1, such as BI2536, GSK461364, and BI6727, have been studied in a wide variety of tumors.^[^ ^41^ ^]^ However, current trials involving PLK1 inhibitors have shown little clinical success.^[^ ^41^ ^]^ We analyzed the relationship between PLK1 and CBX3 and found that PLK1 phosphorylated CBX3 to regulate the sensitivity of CRPC to CDK4/6 inhibitors. The idea of dual inhibitors that target both kinases and bromodomains has been investigated in recent years. The PLK1 inhibitor BI‐2536 and the JAK2 inhibitor TG101209 are highly effective inhibitors of BET bromodomains, exhibiting IC50 values of 0.025 and 0.13 mM, respectively, against BRD4.^[^ ^43^ ^]^ In addition to significant inhibition of PLK1, PLK1 inhibitors can also reduce downstream c‐MYC phosphorylation and weaken the binding of BRD4 to the c‐MYC promoter.^[^ ^44^ ^]^ As a result, dual‐target inhibitors that concurrently inhibit BET and PLK1 have shown promise in increasing the effectiveness of both targets while minimizing toxicity.^[^ ^45^ ^]^ The use of two molecules to inhibit PLK1 and BET seems to induce a synergistic effect via the BRD4 and may reduce toxicity.^[^ ^46^ ^]^ These experimental results show that BRD4 regulates the expression level of CBX3 and that PLK1 regulates the phosphorylation of CBX3. We revealed that the dual BRD4/PLK1 inhibitors partially sensitize PC cells to the anticancer effects of CDK4/6 inhibitors through CBX3. This finding has a certain reference value for the future treatment of CRPC.

## 4 Conclusion

Collectively, this study aimed to identify the genes that are involved in the regulation of CRPC cell sensitivity to BET inhibitors and CDK4/6 inhibitors. Our data demonstrated that CDK4/6 inhibitor treatment increased the expression of CBX3, which was also upregulated in the CDK4/6 inhibitor‐resistant cells. Then, we showed that CBX3 is transcriptionally upregulated by BRD4 and downregulated by BET inhibitors in CRPC cells. Moreover, CBX3 promoted cell cycle progression by binding with RB1 to release E2F1, which decreased the sensitivity of CRPC to CDK4/6 inhibitors. Moreover, we revealed that PLK1 phosphorylated CBX3 to enhance the interaction between RB1 and CBX3. Therefore, we proposed that a dual BRD4/PLK1 inhibitor could increase of CRPC cells to CDK4/6 inhibitors partially through CBX3 (Figure S7H, Supporting Information).

## 5 Experimental Section

### Cell Lines and Cell Culture

The prostate cancer cell lines PC‐3(SC0126), 22RV1(SC0129), and DU145(SC0128) were obtained from Yuchicell Biology Technology (Shanghai, China). C4‐2(SNL‐160) was purchased from Wuhan Shang En Biotechnology Co., Ltd (Wuhan, China). These cells were authenticated using short tandem repeat (STR) profiling and regularly screened for mycoplasma contamination using the PlasmoTest – Mycoplasma Detection Kit (Catalog#: rep‐pt1, InvivoGen, China). All cells were cultured in DMEM (Dulbecco's Modified Eagle Medium) supplemented with 10% fetal bovine serum (FBS) (Catalog#: AC03L055, Shanghai Life‐iLab Biotech, China) and incubated at 37 °C in a 5% CO_2_ environment.

### Chemicals and Reagents

Chemicals used as follows: JQ1 (#S7110), Palbociclib HCl (#S1116), BI‐2536 (#S1190), and Volasertib (#S2235) were obtained from Selleck (Shanghai, China). The cDNA of CBX3, RB1, or BRD4 was cloned into the OmicLinkTM Expression Clone (CMV Promoter) plasmids (Catalog#: EX‐V0006‐M14, GeneCopoeia, USA) for CBX3, RB1, or BRD4 overexpression. The mutant of CBX3 were also generated by the GeneCopoeia. The siRNA and shRNA used were obtained from RiboBio (Guangzhou, China). The sequences of siRNA and shRNA were provided in the Table S1 (Supporting Information).

### Plasmids and siRNA Transfection Infection

The cells were cultured in plates or dishes and subjected to a 12 h period of serum starvation using serum‐free Opti‐MEM medium (Catalog#: 31 985 062, ThermoFisher Scientific, Shanghai, China). Subsequently, the designated siRNA, plasmids or shRNAs and Lipofectamine 2000 (#12 566 014, ThermoFisher Scientific, shanghai, China) were incubated together for 20 min in 1 mL serum‐free Opti‐MEM medium. The resulting mixture was transferred to plates or dishes. After 6 h, the serum‐free Opti‐MEM medium was replaced with complete DMEM medium and the cells were further incubated for a period of 48 h for plasmids or siRNAs, or of 72 h for shRNAs. For the transfection of shRNAs, puromycin was applied to select the positively transfected cells.

### Co‐Immunoprecipitation (Co‐IP) and Western Blot Analysis

For co‐immunoprecipitation (Co‐IP) assay, the cells were harvested and lysed with RIPA buffer (containing 1 mM dithiothreitol (DTT), 100 mmol/l NaCl, and 1 mM MgCl2) (#P0013, Beyotime, China) and then the proteins were extracted. The protein amount was determined by using the BCA method. For Co‐IP, the proteins were added with protein A+G beads (#P2029, Beyotime, China) and IgG (#A7007, Beyotime, China) or a primary antibody, and rotated in the cold room overnight. The next day, the beads were washed 6 times with RIPA buffer, followed by the addition of 60 µL of loading buffer. Finally, the beads were boiled and subjected to Western blot analysis to detect the protein of interest.

For Western blot analysis, proteins were boiled and separated by sodium dodecyl sulfatepolyacrylamide gel electrophoresis (SDS‐PAGE) gel separation. The proteins were transferred onto 0.45 µm polyvinylidene fluoride membranes (Millipore, USA), and incubated with the specific primary antibodies followed by secondary antibodies. Protein signals were detected using the ECL detection reagent (Thermo Fisher Scientific, USA) and ChemiDoc XRS (Bio‐Rad Laboratories, USA). Antibodies used as follows: pRB1‐S795 (#9301,Cell signaling technology, 1:500 dilution), CBX3 (#11650‐2‐AP, Proteintech, 1:1000 dilution), PLK1 (#10305‐1‐AP, Proteintech, 1:1000 dilution), RB1 (#9309, Cell signaling technology, 1:500 dilution), GAPDH (#60004‐1‐Ig, Proteintech, 1:5000 dilution), MCM7 (#11225‐1‐AP, Proteintech, 1:2000 dilution), cleaved caspase 3 (#9661, Cell signaling technology, 1:2000 dilution), BRD4 (#13 440, Cell signaling technology, 1:1000 dilution).

### Quantitative Real‐Time PCR (RT‐qPCR) and Chromatin Immunoprecipitation (ChIP)‐qPCR Analysis

The details of RT‐qPCR were described previously.^[^ ^19^ ^]^ To summarize, total RNA was extracted from cells using TRIzol reagent (ThermoFisher Scientific, USA). The extracted RNA was then subjected to reverse transcription using the PrimeScript RT Reagent Kit (#RR037A) and PCR amplification using the TB Green Fast qPCR Mix (#RR430A) from Takara Bio Inc. (Shigo, Japan) for the RT‐qPCR assay. To conduct ChIP‐qPCR analysis, the ChIP Kit Magnetic – One Step (#ab156907) from Abcam (USA) and the Chromatin Extraction Kit (#ab117152), also from Abcam (USA) were used. The procedures followed were consistent with the previously described methods.^[^ ^20^ ^]^ The sequences of primers were provided in Tables S2 and S3 (Supporting Information).

### Cell Proliferation Assay

The Cell Counting Kit‐8 (CCK‐8) was used to determine the proliferative capacity of cells in vitro as previously reported.^[^ ^21^ ^]^ To summarize, CCK‐8 reagent (#C0037, Beyotime, China) was added to each well containing cells and the absorbance at 450 nm was measured with a microplate reader.

### In Vivo Xenografts Assay

BALB/C‐nu/nu mice (Hunan SJA Laboratory Animal Company), male, 6 weeks old, were individually housed in the animal center of the Second Xiangya Hospital, Central South University. The animals were provided with ad libitum access to food and water without any restriction. For the subcutaneous injection of prostate cancer cells on the left side of the back (5 × 10^6^ cells per mouse), the mice were arbitrarily divided into sub‐groups devoid of blinding or participants. Following injection, Vernier calipers were used to measure the length and width of the tumors every two days, and the tumor volume was calculated utilizing the formula (L × W^2^) / 2. The administration of medication was described in the figure legends, and mice were euthanized at the appropriate time, with the tumors harvested for further research. Animal experiments were approved by the Ethical Committee on Animal Experiments of the Second Xiangya Hospital, Central South University in Changsha, China (Approval No. 20 230 477). The animal experiment complied with the National Institutes of Health guide for the care and use of Laboratory animals (NIH Publications No. 8023, revised 1978).

### Data Mining and Bioinformatics Analysis

The Cancer Genome Atlas (TCGA), Gene Expression Omnibus (GEO) were used for data mining and bioinformatics analysis. In this study, the data generated were publicly available in Gene Expression Omnibus (GEO) at GSE23816625.

### PLK1 Kinase Assay

For in vitro PLK1 kinase assay, the Flag‐CBX3 wild type (WT) or Flag‐CBX3 T60A mutant proteins were transcription and translated in vitro following the manufacture's protocol of TNT T7 Quick Coupled Transcription/Translation System (Promega, # L1170) as previously described.^[^ ^22^, ^23^ ^]^ These proteins were immobilized with protein A+G beads and Flag‐tagged antibody (# AYC01‐100, Shanghai Yoche Biotechnology Co, China) in the cold room overnight. Then, protein A+G beads with Flag‐tagged antibody and in vitro translated proteins were added the buffer (40 mM Tris‐HCl pH 7.4, 20 mM MgCl2, 0.1 mg mL^−1^ BSA and 1 mM DTT), active recombinant human PLK1 protein (#ab271716, Abcam, Shanghai, China), and 50 µM ATP‐γ‐S (#ab138911, Abcam) at 30 °C for 45 min. PNBM/5% DMSO of 2.5 mM were added to the sample at the room temperature for 1 h. The phosphorylated protein was detected by thiophosphate ester antibody (# ab92570, Abcam).

For PLK1 kinase assay in cells, the C4‐2 cells were transfected with indicated constructs or treated with indicated agents, for example Flag‐CBX3 WT or Flag‐CBX3 T60A plus siNC or siPLK1, Flag‐CBX3 WT or Flag‐CBX3 T60A plus vehicle or volasertib for 48 h and added with 50 µM ATP‐γ‐S for other 4 h. Cells were harvested and immunoprecipitated with protein A+G beads and Flag‐tagged antibody in the cold room for 4 h. PNBM/5% DMSO of 2.5 mM were added to the sample at the room temperature for 1 h. The phosphorylated protein was detected by thiophosphate ester antibody.

For in vitro phosphorylation experiment of PLK1, the Glutathione S‐transferase (GST)‐EV, GST‐CBX3 WT or GST T60A plasmids were transfected in the Escherichia coli BL21. The GST‐tagged recombinant proteins were purified by GST‐beads. The HA‐RB1 wild type (WT) proteins were transcription and translated in vitro following the manufacture's protocol of TNT T7 Quick Coupled Transcription/Translation System (Promega, # L1170) as previously described.^[^ ^22^, ^23^ ^]^ These proteins were immobilized with protein A+G beads and HA‐tagged antibody (#AYC02‐100, Shanghai Yoche Biotechnology Co, China). The GST‐beads and h protein A+G beads were mixed and added the buffer (40 mM Tris‐HCl pH 7.4, 20 mM MgCl2, 0.1 mg mL^−1^ BSA and 1 mM DTT and 20 µM ATP) with or without PLK1 protein (#ab271716, Abcam) at 30 °C for 45 min. Then, these beads were boiled and subjected to Western blot analysis.

### Apoptosis Assay

The apoptosis of cells was measured using the caspase 3 activity assay and Annexin V‐FITC/7‐PI assay. In the caspase 3 activity assay, a Caspase‐3 Assay kit (ab39401) from Abcam was utilized. The assay was conducted according to the manufacturer's protocol provided with the kit. For the Annexin v‐FITC/7‐PI assay, cells were stained with Annexin V‐FITC and PI following the manufacturer's instructions for the Annexin V‐FITC apoptosis assay kit (#abs50001, Absin, Shanghai, China). Following incubation at room temperature for a duration of 15 min, the cells were examined via a flow cytometer and subsequently analyzed utilizing the FlowJo analytical software.

### Immunofluorescence Assay

Cells were incubated with 1,1′‐dioctadecyl‐3,3,3′,3′‐tetramethylindodicarbocyanine,4‐chlorobenzenesulfonate Salt (DiD, Far‐red Plasma Membrane Fluorescent Probe, Beyotime, C1039, China) for 20 min to label the cell membrane. Then, the cells were fixed with paraformaldehyde for 15 min and permeabilized with 0.2% Triton X‐100 for 10 min. Next, the cells were incubated with primary antibodies at 4 °C overnight. The following day, the cells were incubated with fluorescent secondary antibodies (Beyotime, China) for 1 h, and washed three times with PBS buffer. Then DAPI (Beyotime, China) was used to stain cell nuclei for 10 min. After washing with PBS buffer for three times, the samples were analyzed through confocal microscopy.

### Statistical Analysis

The experimental data were presented as the mean ± standard error of the mean (mean ± SEM). The sample size (n) for each statistical analysis was indicated in the figure legends. Statistical significance was calculated using GraphPad Prism 9 software. For the comparison of differences between two groups, the unpaired two‐sided Student's t‐test was employed. For comparisons involving more than two groups, one‐way analysis of variance (ANOVA) was performed, followed by Turkey's multiple comparisons post hoc test. Two‐way ANOVA followed by Turkey's multiple comparisons post hoc test was applied to comparison more than two groups with different time points. Two‐way ANOVA followed by Šídák's multiple comparisons test was applied to comparison two group or more than two groups with concentration of Palbociclib treatment in cells. Statistically significant differences were considered at p < 0.05 (*), p < 0.01 (**), and p < 0.001 (***), while p > 0.05 was considered not significant (ns).

### Ethics Approval and Consent to Participate

The study was conducted in accordance with the principles of the Declaration of Helsinki principles. It was approved by the Animal Use and Care Committees at the Second Xiangya hospital, Central South University.

## Conflict of Interest

The authors declare no conflict of interest.

## Author Contributions

H.L., C.Y., and R.Z. contributed equally to this work. H.L., C.Y., R.Z., and Y.S. performed methodology; R.Z., J.W., and W.X. performed formal analysis; W.X. performed conceptualization; B.Y. and X.J. performed investigation; X.J. performed project administration.

## Supporting information

Supporting Information

## Data Availability

The data that support the findings of this study are available from the corresponding author upon reasonable request.
