# Supplementary material for: Targeting CBX3 with a Dual BET/PLK1 Inhibitor Enhances the Antitumor Efficacy of CDK4/6 Inhibitors in Prostate Cancer
Source: Adv Sci (Weinh). 2023 Nov 10;10(36):2302368. doi: 10.1002/advs.202302368 (PMC10754129; doi:10.1002/advs.202302368)
Supplement: Supplementary file 1 — Supporting Information [file ADVS-10-2302368-s001.pdf]

ADVANCED SCIENCE
Open Access

Supporting Information

for *Adv. Sci.*, DOI 10.1002/advs.202302368

Targeting CBX3 with a Dual BET/PLK1 Inhibitor Enhances the Antitumor Efficacy of CDK4/6 Inhibitors in Prostate Cancer

Huaiyuan Liang, Chunguang Yang, Ruijiang Zeng, Yingqiu Song, Jianxi Wang, Wei Xiong, Binyuan Yan and Xin Jin**

Targeting CBX3 with a dual BET/PLK1 inhibitor enhances the antitumor efficacy of CDK4/6 inhibitors in prostate cancer

Huaiyuan Liang, Chunguang Yang, Ruijiang Zeng, Yingqiu Song, Jianxi Wang, Wei Xiong, Binyuan Yan, Xin Jin

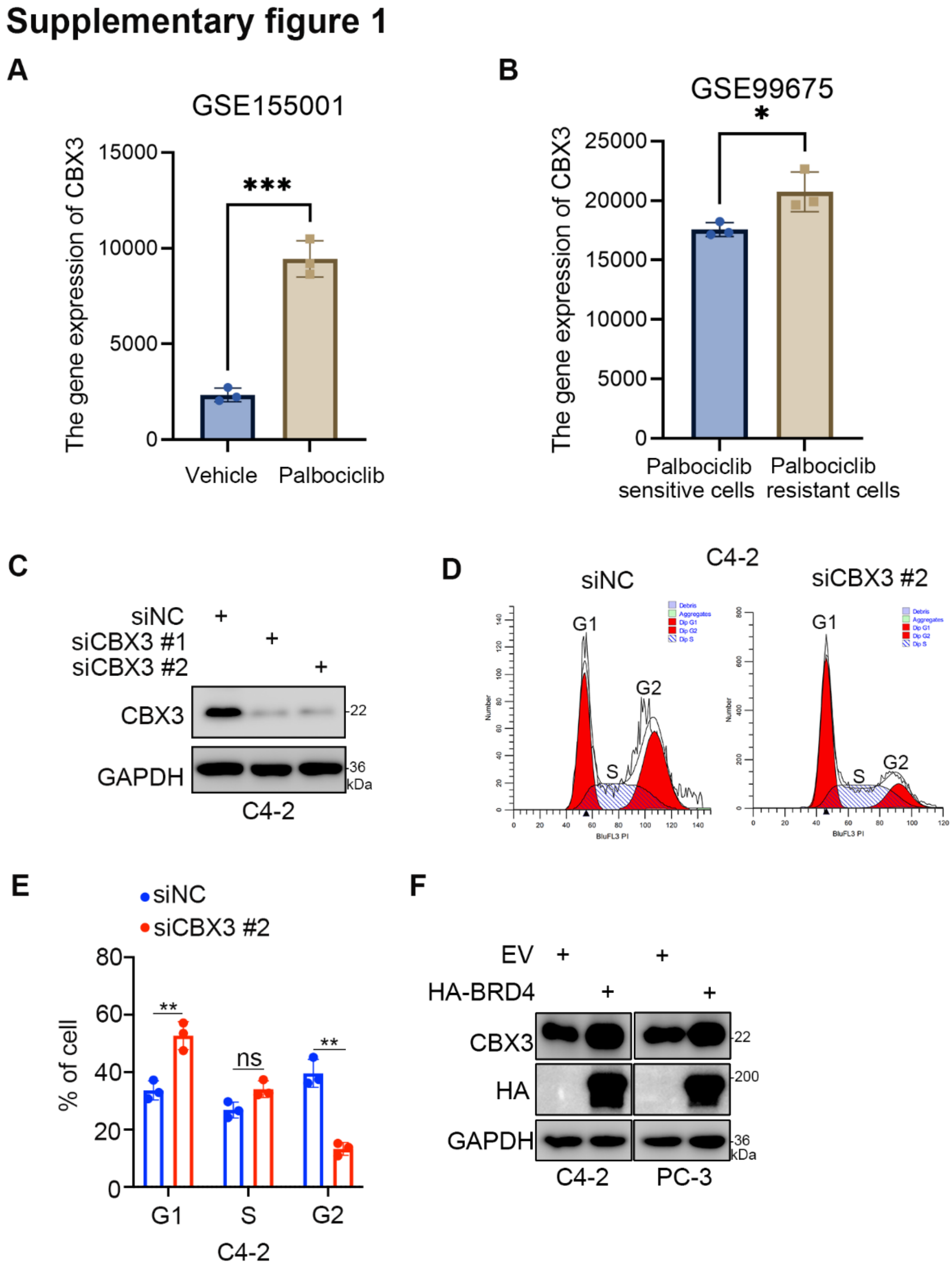

Supplementary figure 1

A and B, analysis of the GSE155001 and GSE99675 to detect the mRNA level of CBX3 in corresponding prostate cancer cells. *, $P < 0.05$; ***, $P < 0.001$. For GSE155001 (panel A), PC-3 cells were treated with vehicle (DMSO) or 5 μM palbociclib for 24 hours. For GSE99675 (panel B), palbociclib resistant cells were generated from LNCaP cells via sustained treatment with 0.5 μM palbociclib and kept under palbociclib-selection to maintain the cells for 2~3 months. Parental cells were cultured in parallel in standard growth media. The palbociclib sensitive cells were defined as parental cells initiated treated with 0.5 μM palbociclib. **C-E,** C4-2 cells were transfected with indicated siRNAs for 48 h. Cells were harvested for Western blot (C) and cell cycle analysis (D and E). Data presents as mean ± SEM with three replicates. Ns, not significant; **, $P < 0.01$. **F**, C4-2 and PC-3 cells were transfected with indicated plasmids for 48 h. Cells were harvested for Western blot analysis.

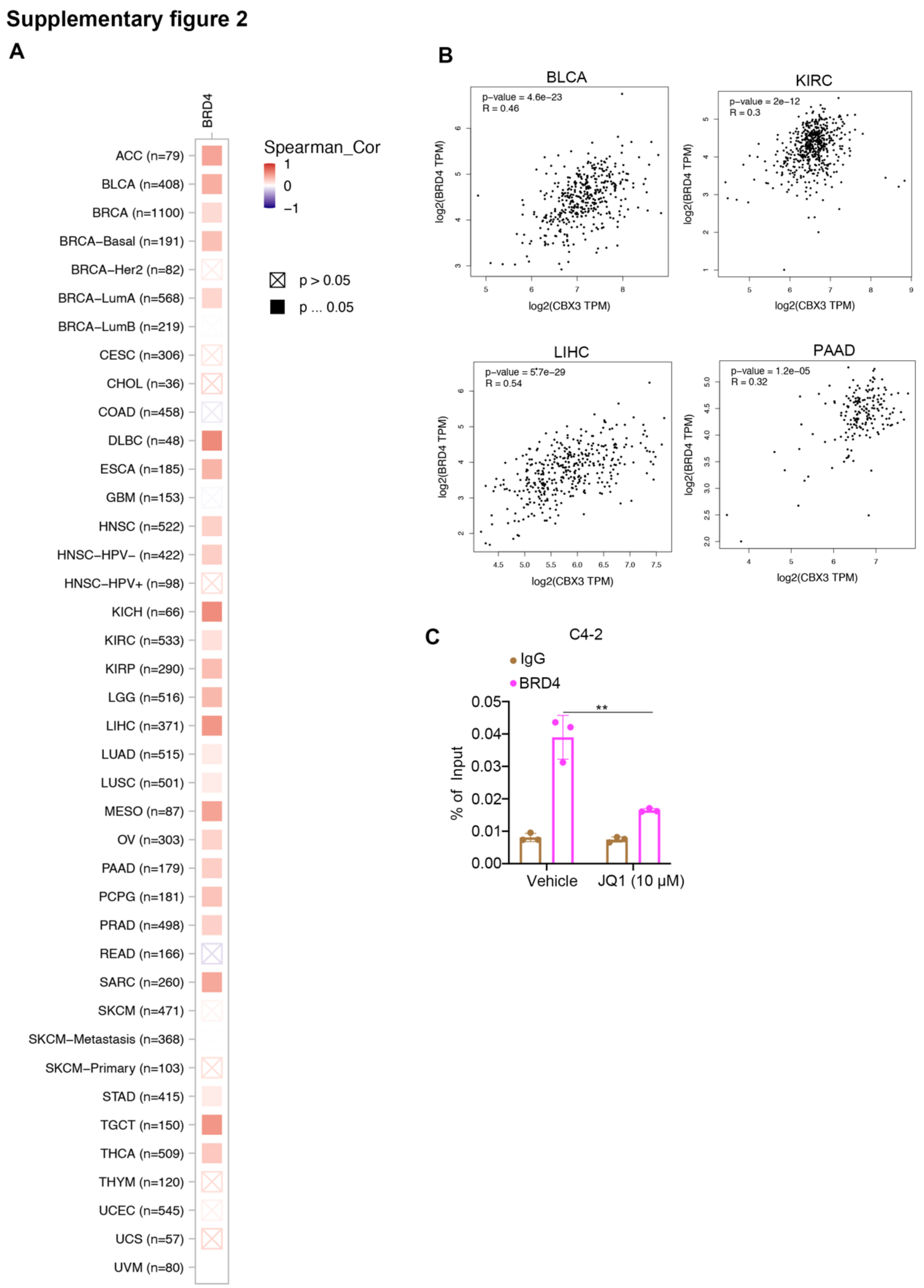

Supplementary figure 2.

A and B, analysis of the TCGA dataset to show the correlation between BRD4 and CBX3. **C**, C4-2 cells were treated with or without 10 μM JQ1 for 24 h. Cells were

harvested for ChIP-qPCR assay. Data presents as mean ± SEM with three replicates. Ns, not significant; **, P < 0.01.

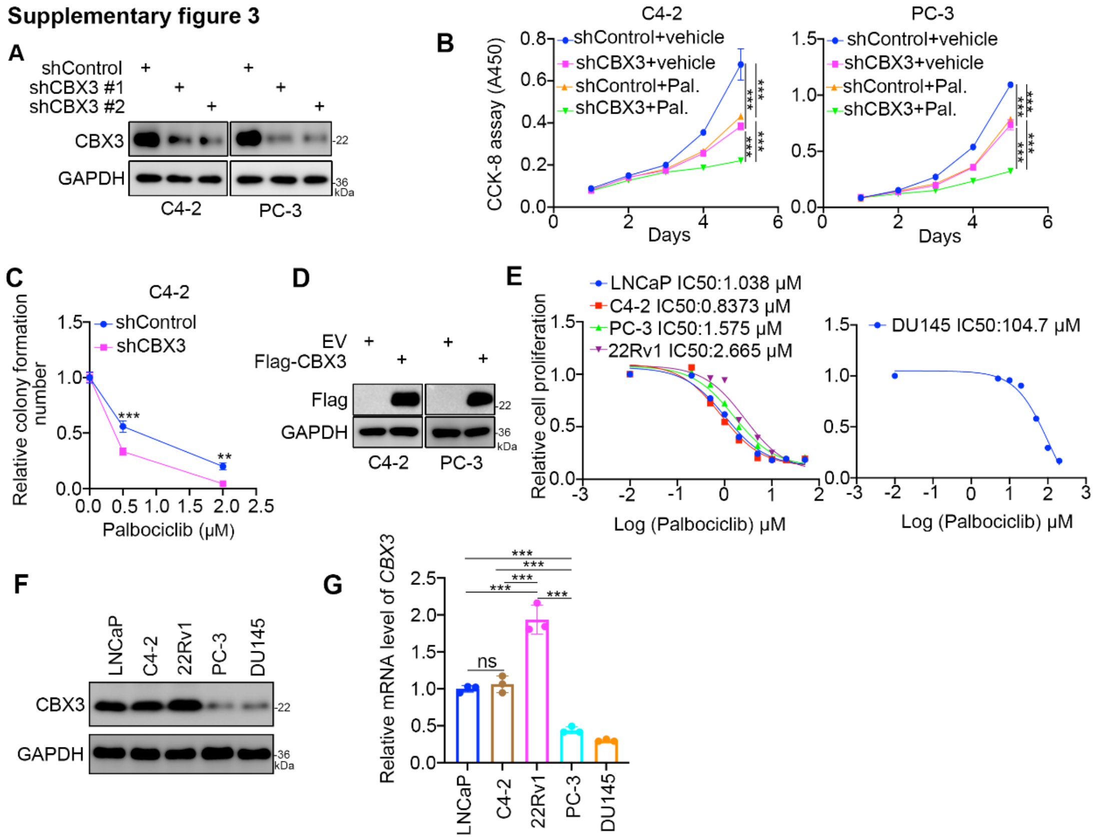

Supplementary figure 3.

A, C4-2 and PC-3 cells were transfected with indicated shRNAs for 72 h. Cells were harvested for Western blot analysis. **B**, C4-2 and PC-3 cells were transfected with indicated shRNAs for 72 h. Cells were treated with or without 2μM palbociclib and subjected to CCK-8 assay. Data presents as mean ± SEM with three replicates. Ns, not significant; ***, P < 0.001. **C**, C4-2 cells were transfected with indicated shRNAs for 72 h. Cells were treated with a serial dose (0, 0.5, 2 μM) of palbociclib and subjected to colony formation assay. Data presents as mean ± SEM with three replicates. **, P < 0.001; ***, P < 0.001. **D**, C4-2 and PC-3 cells were transfected with indicated plasmids

for 48 h. Cells were harvested for Western blot analysis. **E**, CCK-8 assay were used to detect the IC50 values of palbociclib in different prostate cancer cells. **F** and **G**, Western blot assay and RT-qPCR analysis were used to detecte protein and mRNA levels of CBX3 in different prostate cancer cells. Data presents as mean ± SEM with three replicates. NS, not significant; ***, P < 0.001.

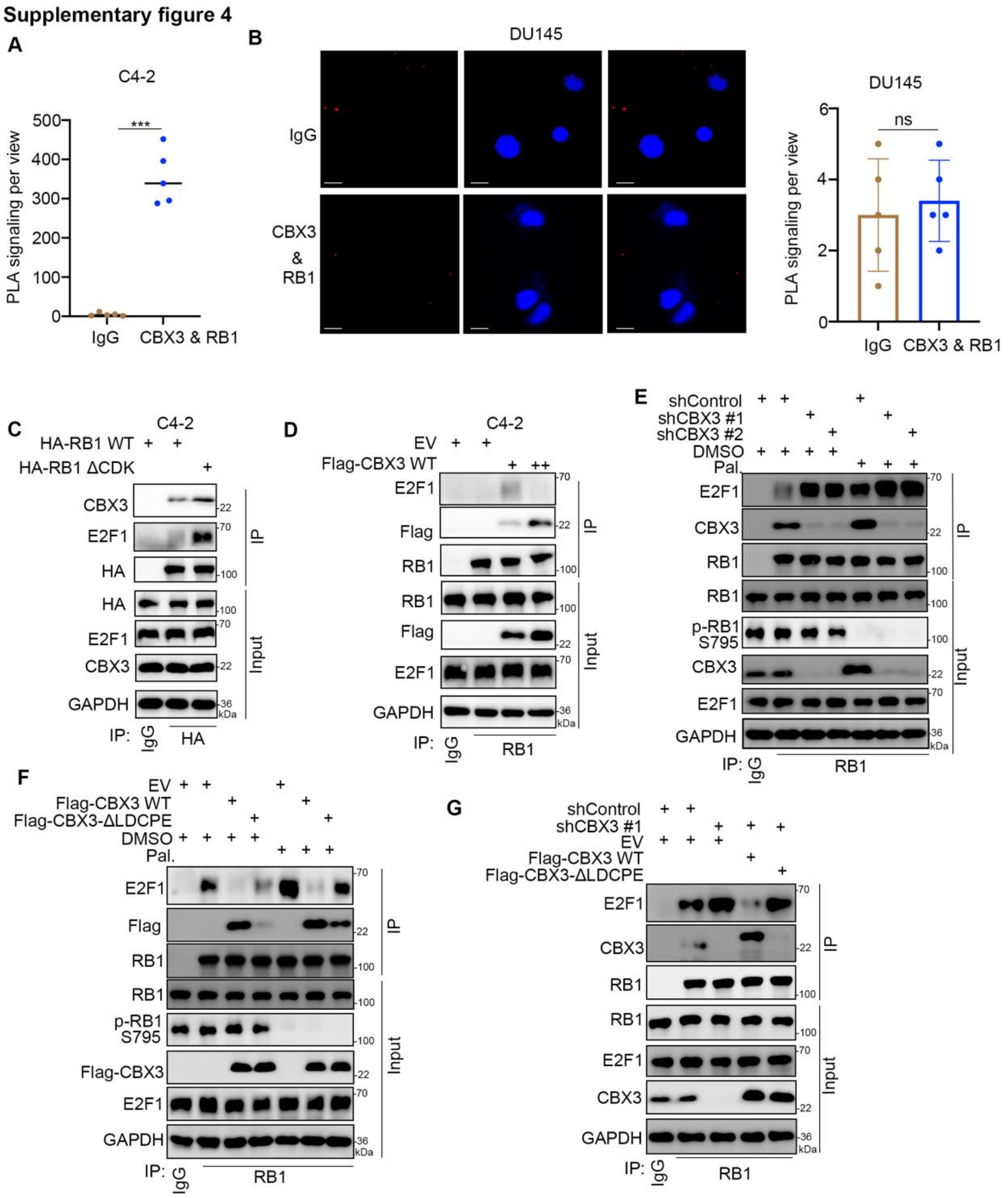

Supplementary figure 4.

A-B, the PLA assay was performed in C4-2 and DU145 cells by using the indicated antibodies. Data presents as mean ± SEM with five replicates. NS, not significant; ***, $P < 0.001$. **C and D,** C4-2 cells were transfected with indicated plasmids for 48h. Cells were harvested for IP assay and Western blot analysis. **E**, C4-2 cells were transfected with indicated shRNAs for 48h. Then, these cells were treated with or without 1μM palbociclib for other 24h. Cells were harvested for IP assay and Western blot analysis. **F**, C4-2 cells were transfected with indicated plasmids for 48h. Then, these cells were treated with or without 1 μM palbociclib for other 24h. Cells were harvested for IP assay and Western blot analysis. **G**, C4-2 cells were transfected with indicated plasmids for 48h or shRNAs for 72h. Cells were harvested for IP assay.

Supplementary figure 5

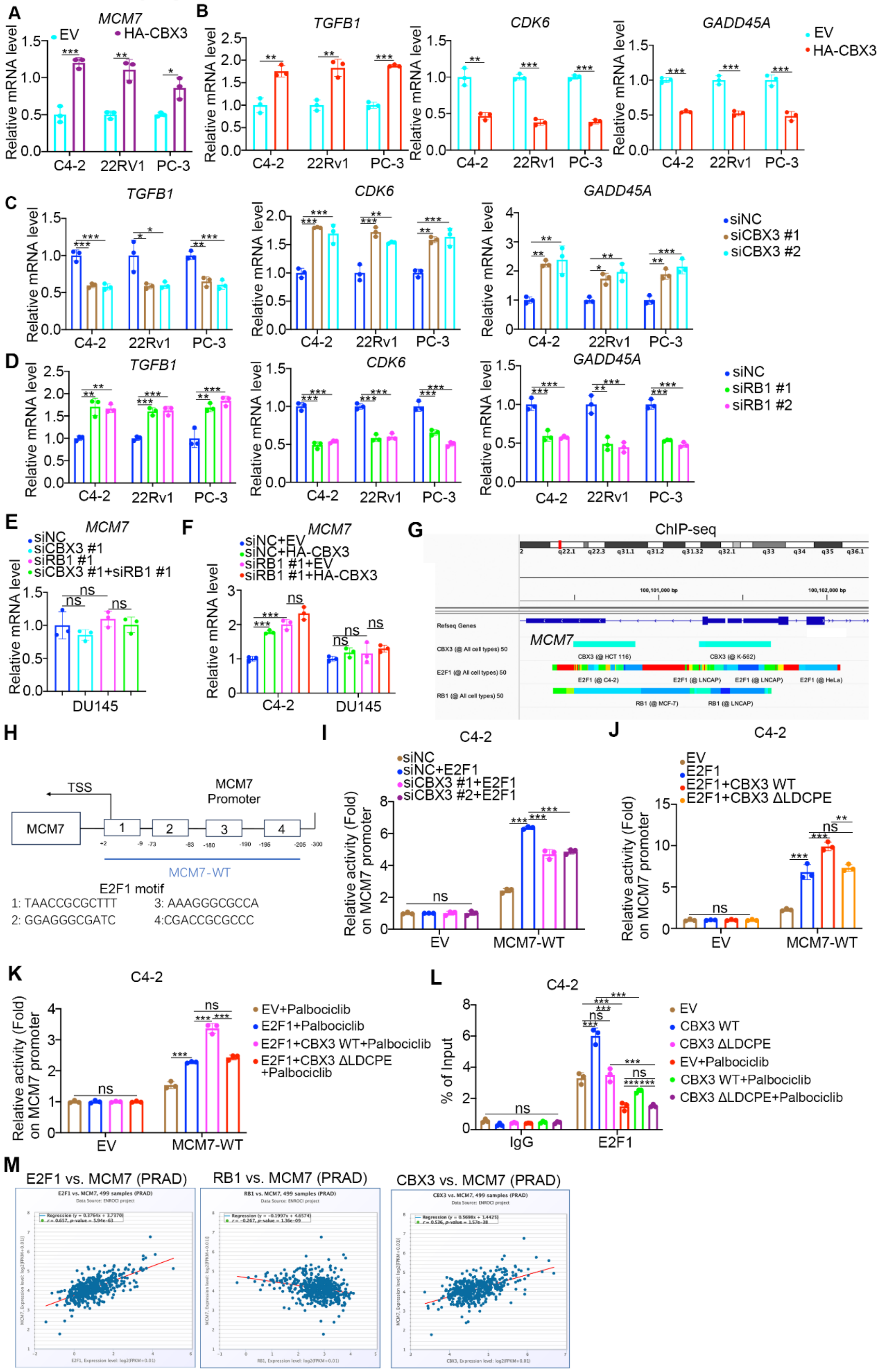

Supplementary figure 5.

A-F, C4-2, 22Rv1 and PC-3 cells were transfected with indicated siRNAs or plasmids for 48h. Cells were harvested for RNA-seq analysis. Data presents as mean ± SEM with three replicates. Ns, not significant; *, P < 0.05, **, P < 0.01, ***, P < 0.001. **G**, the ChIP-seq of CBX3, E2F1 and RB1 on the promoter region of *CBX3*. **H**, diagram demonstrated that the E2F1 motif in the MCM7 promoter. TSS, transcriptional start site. **I-J**, C4-2 cells were transfected with empty vector (EV), GV592-MCM7, and indicated siRNAs or plasmids for 48h. Cells were harvested for luciferase reporter assay. Data presents as mean ± SEM with three replicates. Ns, not significant; **, P < 0.01, ***, P < 0.001. **K**, C4-2 cells were transfected with empty vector (EV), GV592-MCM7, and indicated plasmids for 48h. Then, cells were treated with or without 1 μM palbociclib for other 24h. Cells were harvested for luciferase reporter assay. Data presents as mean ± SEM with three replicates. Ns, not significant; ***, P < 0.001. **L**, C4-2 cells were transfected with indicated plasmids for 48h. Then, cells were treated with or without 1 μM palbociclib for other 24h. Cells were harvested for ChIP-qPCR. Data presents as mean ± SEM with three replicates. Ns, not significant; ***, P < 0.001.**M**, the correlation between CBX3 and MCM7, E2F1 and MCM7, and RB1 and MCM7 in the TCGA-PRAD dataset.

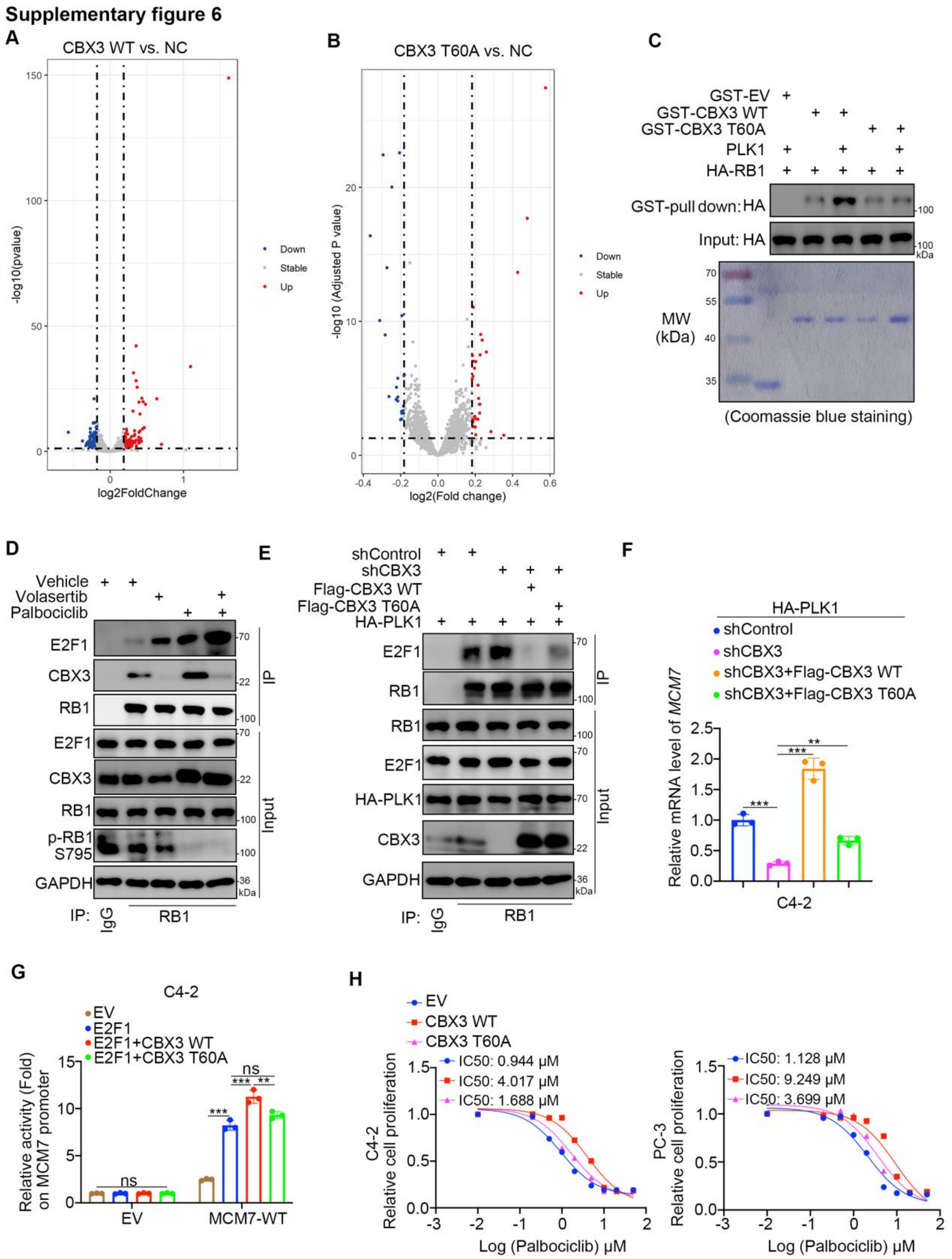

Supplementary figure 6.

A and B, C4-2 cells were transfected with empty vector, CBX3 wild type and CBX3 T60A plasmids for 48 h. These cells were subjected to RNA-seq analysis. **C**, GST-pulled down assay and PLK1 kinase assay was performed by using the recombinant

protein of HA-RB1, PLK1, CBX3 WT and CBX3 T60A. **D,** C4-2 cells were treated with 1 μM volasertib or 1 μM palbociclib for 24 h. Cells were harvested for IP assay**.** **E and F**, C4-2 cells were transfected with indicated plasmids or shRNAs for 72h. Cells were harvested for IP assay and RT-qPCR assay. Data presents as mean ± SEM with three replicates. **, P < 0.01, ***, P < 0.001. **G**, C4-2 cells were transfected with empty vector (EV), GV592-MCM7, and indicated plasmids for 48h. Cells were harvested for luciferase reporter assay. Data presents as mean ± SEM with three replicates. Ns, not significant; **, P < 0.01; ***, P < 0.001. **H**, C4-2 and PC-3 cells were transfected with indicated plasmids for 48 h. Then, cells were treated with a serial dose of palbociclib for 24 h and subjected to CCK8 assay.

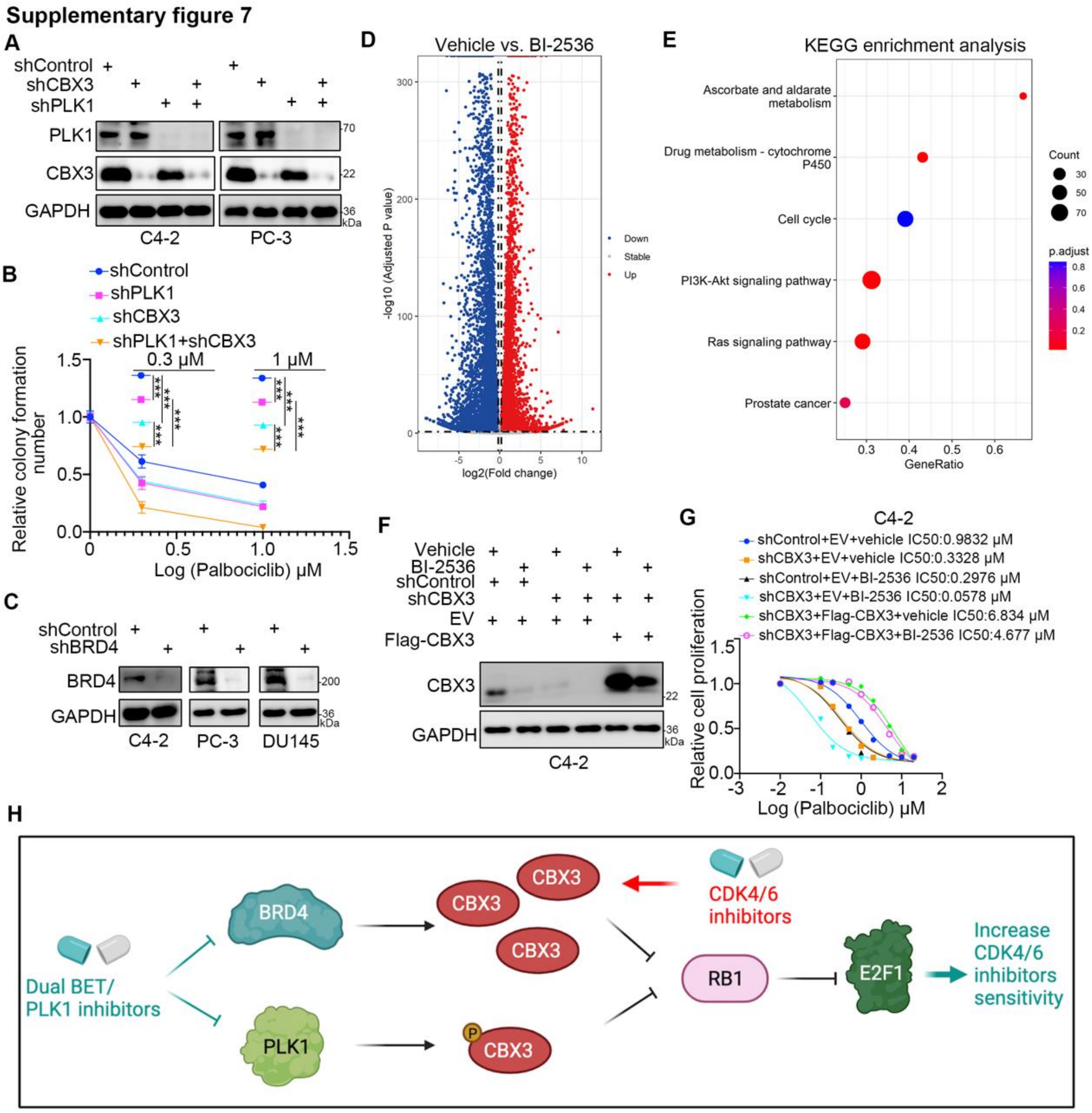

Supplementary figure 7.

A, C4-2 and PC-3 cells were transfected with indicated shRNAs for 72 h. Cells were subjected to Western blot analysis**. B,** C4-2 cells were transfected with indicated shRNAs for 72 h. Cells were treated with a serial dose of palbociclib and subjected to colony formation assay. Data presents as mean ± SEM with three replicates. ***, P < 0.001. **C,** C4-2, PC-3 and DU145 cells were transfected with indicated shRNAs for 72 h. Cells were subjected to Western blot analysis. **D and E,** C4-2 cells were treated with 10 μM BI-2536 for 24 h. Cells were subjected to RNA-seq analysis. **F and G**,

C4-2 cells were transfected with indicated shRNAs or plasmids for 72 h. Then, cells were treated with or without 10 μM BI-2536 for another 24 h. Cells were subjected to Western blot analysis and CCK8 assay**. H,** a model depicting that a dual BRD4/PLK1 inhibitor could increase the sensitivity of CDK4/6 inhibitors partially through the CBX3/RB1/E2F1 axis in CRPC cells.

Supplementary Material and Methods

Glutathione S-transferase pull-down assay

Cells were lysed with 1 × RIPA lysis buffer (P0013B, Beyotime, shanghai, China) for 30 minutes at 4 ℃. Glutathione S-transferase (GST) fusion proteins were immobilized on BeyoMag™ Anti-GST Magnetic Beads (P2138, Beyotime, shanghai, China). After washed with 1 × RIPA lysis buffer, the beads were incubated with cell lysates for 4 hours. The beads were then washed four times with 1 × RIPA lysis buffer and resuspended in loading buffer. The bound proteins were subjected to SDS/PAGE and Western blotting.

Liquid chromatography-tandem mass spectrometry/mass spectrometry analysis

For the mass spectrometry analysis of ZDHHC2, the cell lysates of 293T cells were collected and immunoprecipitated with IgG antibodies or ZDHHC2 and protein A+G agarose beads (#P2012, Beyotime, Shanghai, China) at 4 °C. The mass spectrometry analysis was conducted by SpecAlly Life Technology Co., Ltd, Wuhan, China.

Sample preparation: the beads samples obtained from immunoprecipitation experiment were washed three times with pre-cooled PBS buffer to remove the remaining detergent.

Then beads samples were incubated in the reaction buffer (1% SDC/100 mM Tris-HCl, pH 8.5/10 mM TCEP/40 mM CAA) at 95 °C for 10 min for protein denaturation, cysteine reduction and alkylation. The eluates were diluted with equal volume of H2O and subjected to trypsin digestion overnight by adding 1 μg of trypsin at 37 °C. The peptide was purified using self-made SDB desalting columns. The eluate was vacuum dried and stored at -20 °C for later use.

LC-MS/MS Detection: LC-MS/MS data acquisition was carried out on a Q Exactive HF-X mass spectrometer coupled with an Easy-nLC 1200 system (both Thermo Scientific). Peptides were first loaded onto a C18 trap column and then eluted into a C18 analytical column (75 μm × 250 mm, 3 μm particle size, 100 Å pore size, Acclaim PepMap C18 column, Thermo). Mobile phase A (0.1% formic acid) and mobile phase B (80% ACN, 0.1% formic acid) were used to establish a 120 min gradient. A constant flow rate was set at 300 nL/min. For DDA mode analysis, each scan cycle consisted of one full-scan mass spectrum (R = 120 K, AGC = 3e6, max IT = 50 ms, scan range = 350–1800 m/z) followed by 20 MS/MS events (R = 60 K, AGC = 2e5, max IT = 110 ms). HCD collision energy was set to 32. Isolation window for precusor selection was set to 1.6 Da. Former target ion exclusion was set for 40 s.

GSEA for the key gene

GSEA: KIRC patients were first divided into two groups according to the median expression level of the key gene. Then, differential expression analysis was applied between the high and low expression groups. Input genes for GSEA were sorted by

their logFC values. Signaling pathways activated or suppressed by the key gene were decided by the normalized enrichment score (NES) value derived from GSEA.

ssGSEA: ssGSEA was used to calculate separate enrichment scores for each pairing of a KIRC sample and KEGG gene set. The ssGSEA score was further rescaled by min-max normalization method. Correlation analysis was performed between expression values of key gene and NES of signaling pathways.

Luciferase reporter assay

GV592-MCM7 promoter reporter plasmids WT (- 207bp) were constructed into GV592 backbone (MCS-SV40-firefly_luciferase-PolyA-Tk-Renila_Luciferase-PolyA) by GENECHEM (Shanghai, China). The GV592-MCM7 promoter reporter plasmid was transfected into cells by using the Lipofectamine 2000 (Thermo Fisher Scientific, USA). Forty-eight hours post transfection, cells harvested and tested with the Dual Luciferase Reporter Gene Assay Kit (Beyotime, RG027). All experiments were performed in three times.

Establishing the palbociclib-resistant C4-2 cells

A palbociclib-resistant cell model was generated by continuous treatment of C4-2 cells with 0.5 μM PD, and the success of the construct was tested by IC50 value after about 3 months.

Table S1. The siRNA and sgRNA sequences.

siCBX3 #1	5'- TGGGAAAGTGGAATATTTCCTGA-3'
siCBX3 #2	5'- GACAGCAAATCAAAGAAGAAAAG-3'
siBRD4 #1	5'- AGGACTTCAACACTATGTTTACA-3'
siBRD4 #2	5'- AAGACAAGAAGGAAAAGAAAAAA-3'
siPLK1 #1	5'- GTGAAAATAGGGGATTTTGGACT-3'
siPLK1 #2	5'- CTGCTTAATGACGAGTTCTTTAC-3'
shCBX3 #1	5′- CCGGCTGGCGAAAGAGGCAAATATGCTCGAGCATATTTGCCTCTTTCGCCAGTTTTTG-3′
shCBX3 #2	5′- CCGGCGACGTGTAGTGAATGGGAAACTCGAGTTTCCCATTCACTACACGTCGTTTTT-3′
shBRD4	5’- CCGGCCTGGAGATGACATAGTCTTACTCGAGTAAGACTATGTCATCTCCAGGTTTTT-3’
shRB1 #1	5′- CCGGGTGCGCTCTTGAGGTTGTAATCTCGAGATTACAACCTCAAGAGCGCACTTTTTG -3′
shRB1 #2	5′- CCGGCAGAGATCGTGTATTGAGATTCTCGAGAATCTCAATACACGATCTCTGTTTTTG -3′

Table S2. The primer sequences for RT-qPCR.

Gene （Human）	Forward primer (5′ - 3′)	Reverse primer (5′ - 3′)
GAPDH	ATGACAATGAATACGGCTACAGCA	GCAGCGAACTTTATTGATGGTATT
MCM7	CCCTCGTAGTATCACGGTGC	AGCCCCAGACTCATCATCCT
CBX3	GAGATGCTGCTGACAAACCA	TATTTGCCTCTTTCGCCAGC
TGFB1	ACCTGCCACAGATCCCCTAT	CCGGTAGTGAACCCGTTGAT
CDK6	TCTGATTACCTGCTCCGCGA	CCTCCTCTTCCCTCCTCGAA
GADD45A	AGAAGACCGAAAGCGACCC	GTTGATGTCGTTCTCGCAGC

Table S3. The primer sequences for ChIP-qPCR.

Gene （Human）	Forward primer (5′ - 3′)	Reverse primer (5′ - 3′)
CBX3	GCCTTCCCTTTTTGTTTTCC	ACCACTTTCAGCAGCGAACT
MCM7	GCTCAGAGGTCTTGCTCCTG	CCTCCCCGTCACTCATTCTA
